# miR-181a modulates circadian rhythm in immortalized bone marrow and adipose derived stromal cells and promotes differentiation through the regulation of PER3

**DOI:** 10.1038/s41598-018-36425-w

**Published:** 2019-01-22

**Authors:** Matthew Knarr, Anil Belur Nagaraj, Lily J. Kwiatkowski, Analisa DiFeo

**Affiliations:** 1 Case Comprehensive Cancer Center, Case Western Reserve University, Cleveland, OH 44106 USA; 2 Rogel Cancer Center, The University of Michigan, Ann Arbor, MI USA; 3 Department of Obstetrics and Gynecology, The University of Michigan, Ann Arbor, MI USA; 4 Department of Pathology, The University of Michigan, Ann Arbor, MI USA; 5 Present Address: Laboratory Medicine and Pathology, Mayo Clinic, Rochester, MN 55902 USA

## Abstract

miRNAs are important regulators of diverse cellular processes including proliferation, apoptosis, and differentiation. In the context of bone marrow derived stromal cell and adipose derived stromal cell differentiation, miRNAs are established regulators of both differentiation or stemness depending on their target. Furthermore, miRNA dysregulation can play a key role in various disease states. Here we show that miR-181a is regulated in a circadian manner and is induced during both immortalized bone marrow derived stromal cell (iBMSC) as well as primary patient adipose derived stromal cell (PASC) adipogenesis. Enhanced expression of miR-181a in iBMSCs  and PASCs produced a robust increase in adipogenesis through the direct targeting of the circadian factor period circadian regulator 3 (PER3). Furthermore, we show that knocking down endogenous miR-181a expression in iBMSC has a profound inhibitory effect on iBMSC adipogenesis through its regulation of PER3. Additionally, we found that miR-181a regulates the circadian dependency of the adipogenesis master regulator PPARγ. Taken together, our data identify a previously unknown functional link between miR-181a and the circadian machinery in immortalized bone marrow stromal cells and adipose derived stromal cells highlighting its importance in iBMSC and ASC adipogenesis and circadian biology.

## Introduction

Investigating regulation of cell fate determination and differentiation in adult stromal cell populations is a key component necessary to understanding a number of clinically relevant pathologies and to develop effective cell based therapies^1–3^. Of particular interest are what we will refer to as tissue-specific stromal cells with adiopogenic differentiation capacity which until recently have been categorized under the umbrella term of “mesenchymal stromal/stem cells”. Mounting evidence has contributed to the argument that “mesenchymal stem cell” is a generalized misnomer for a wide variety of stromal cell populations each of which have unique functional characteristics in terms of multipotency (the ability to differentiate into a limited subset of cell types), self-renewal (the ability of explanted cells to reconstitute cells *in vivo* that are identical in their phenotype and potency), immunophenotype, and immunomodulatory properties^4,5^. Recent studies have shown that “mesenchymal stem/stromal cells” isolated from different tissue sources have very different gene expression profiles and differentiation capacities *in vivo*^6^. Thus, when we speak about tissue specific stromal cells with adipogenic differentiation capacity we are explicitly referring to two different populations of stromal cells; bone marrow stromal cells and adipose derived stromal cells. Bone marrow stromal cells (BMSCs) are stromal cells that reside on the outer surface of bone marrow sinusoids as part of the perivascular stromal compartment. BMSCs possess both multipotency and self-renewal and are capable of generating a complete heterotopic bone or bone marrow organ (ossicle) *in vivo*, including perivascular stromal cells with similar phenotypes and properties as the originally explanted cell^4,7^. Adipose derived stromal cells (ASCs) are adipocyte progenitor cells that are part of the stromal compartment surrounding blood vessels (either as mural or adventitial cells) within adipose tissue^8–11^. ASCs do not possess the same degree of multipotency (requiring BMP stimulation to undergo osteogenic differentiation) nor self-renewal (evidence that ASCs can give rise to all the cells in the perivascular adipose compartment has not yet emerged)^8,11–14^. The commonality between BMSCs and ASCs is that both possess the ability to differentiate into adipocytes *in vivo*. Adipogenic differentiation of BMSCs and ASCs is a key fixture not only in normal biology but also certain pathological processes. Of these, pathogenic adipogenesis of BMSCs and ASCs is a common, long-term pathology that can contribute to bone marrow adiposity (in the case of BMSCs) and obesity (in the case of ASCs). BMSCs and ASCs contribute to these pathologies based on changes in their surrounding microenvironment which shifts the balance of their differentiation potential to favor adipogenesis. In the case of bone marrow adiposity, both local and systemic factors can act in either an autocrine/paracrine or endocrine manner to affect BMSCs within the bone marrow compartment. These pathologic changes in the microenvironment facilitate a shift in the phenotypic behavior of the BMSCs that inhibits osteogenesis and promotes adipogenesis thus inhibiting bone formation. Similarly, within the adipose tissue of obese individuals an altered microenvironment results in the increased recruitment and adipogenic differentiation of resident perivascular ASCs. This process acts as both cause and consequence in adipocyte hyperplasia. This in turn contributes to a variety of complications including dyslipidemia, hypertension, and type II diabetes.

The process of adipogenesis is tightly regulated and consists of two major stages; the commitment phase where the BMSCs and ASCs become restricted in their differentiation potential to the adipocyte lineage, and the differentiation phase where the committed pre-adipocytes undergo the changes necessary to become functional adipocytes^15,16^. A multitude of factors can act to either promote or repress adipogenic BMSC/ASC differentiation at the molecular, cellular, and physiological level^15,17,18^. Of these various factors, microRNAs (miRNAs) have a large role in controlling BMSC and ASC adipogenesis with several miRNAs acting as either promoters or inhibitors at different spatiotemporal levels of the adipogenic process^19,20^. miRNAs are small, non-coding 19–21 nt RNA molecules that repress gene expression at the post-transcriptional level by binding to the 3′UTR of their target mRNAs to facilitate either translation inhibition and/or mRNA degradation. miRNAs are apt regulators of BMSC and ASC adipogenesis as they have the ability to target the expression of multiple genes simultaneously in a cell and can act as master regulators of gene expression networks in a variety of processes such as cell proliferation, differentiation, and apoptosis. Another key signaling molecule group that influence BMSC/ASC adipogenesis both in the normal, as well as pathogenic, context are circadian factors that are part of the circadian clock^21,22^. The circadian rhythm is a timing mechanism that functions to maintain homeostasis by synchronizing physiological processes. Importantly, circadian coordination ensures proper functioning of processes where BMSC/ASC adipogenesis is involved including bone remodeling and metabolism^23–26^. Circadian rhythm itself consists of a central clock mechanism located in the suprachiasmatic nucleus that responds to environmental cues such as light/dark cycles and feeding patterns. The central clock then entrains the peripheral clocks found in cells (including BMSCs and ASCs) throughout the body^27,28^. A number of circadian machinery components including BMAL1, Nocturnin, PER2, and PER3 have been experimentally shown to affect adipogenesis in bone^23–25^ and adipose^23,29–32^ tissues. Several studies have shown that the PER genes repress BMSC and ASC adipogenesis. In particular, the PER3 gene appears to play a dominant role in the BMSC/ASC adipogenic process by regulating the transcription and protein stability of PPARG. A recent *in vivo* study has highlighted the role of PER3 as a crucial regulator of both the adipogenesis and peripheral circadian clock of ASCs^33^. However, the factors that regulate PER3 in the context of both BMSC/ASC adipogenesis and circadian rhythm have not been completely elucidated.

microRNA-181a (miR-181a) is part of a four member family of miRNAs (miR-181a-d) initially identified in an early computational screen of the human genome for conserved miRNAs^34^. miR-181a has a number of roles in various biological processes including immune development, cancer, and metabolism^35–38^. One of the most intriguing aspects of miR-181a is its ambivalence in acting as a driver of differentiation or stemness depending on the biological context it is acting in. This ability to tip the balance of cell fate toward a more or a less differentiated state is critical in dictating how miR-181a affects a cell by acting to either promote or prevent a pathological process. In cancer biology, miR-181a has been reported to promote cancer progression and recurrence by driving epithelial-mesenchymal transition (EMT) as well as stem-like properties associated with the cancer stem cell phenotype^39,40^. Conversely, in normal physiological systems miR-181a has a critical role in promoting the differentiation and maturation of several cell types including NK, B, and T cells^41–43^. However, its role in the regulation of BMSC/ASC differentiation has not been well characterized.

In this study we investigated the role of miR-181a in BMSC/ASC function using two different cell lines (immortalized bone marrow derived stromal cells and primary visceral adipose derived stromal cells), and whether it affects BMSC/ASC differentiation. Interestingly, we found that endogenous expression of miR-181a was induced during adipogenic differentiation of both immortalized BMSCs and primary ASCs and its enhanced expression produced a robust increase in BMSC/ASC adipogenesis. We found that miR-181a directly targets period circadian clock 3 (PER3) a core regulator of BMSC/ASC adipogenesis circadian rhythm. In addition, we found that miR-181a was regulated in a circadian fashion and could modulate the circadian rhythm of both PPARG and PER3 in BMSCs.

## Materials and Methods

### Cell Culture, Differentiation and Synchronization

Immortalized bone marrow derived Scp-1 cells (iBMSCs) were a generous gift obtained from the lab of Dr. Matthias Schieker (University of Munich). The Scp-1 cells were isolated and immortalized as previously described in^44^. For all experiments Scp-1 cells between passages 80–90 were used. PASC-1 cells were primary ASCs isolated from visceral adipose tissue and purchased from ATCC (ATCC® Number: PCS-500-011™). For PASC-1 cells all experiments were conducted between passages 0–6. Both PASC-1 and Scp-1 cells were maintained in minimum essential medium alpha (αMEM) (Gibco) supplemented with 10% FBS (Denville Scientific) and 0.6% (v/v) penicillin/streptomycin antibiotic. For adipogenic differentiation, iBMSCs or ASCs were seeded in 6 well plates (3 × 10^5^ cells/well) or 10 cm plates (5 × 10^6^ cells/well) and were grown to confluency. Once the iBMSCs or ASCs were confluent the cells were washed 2X in PBS (Corning) and adipogenic induction media (containing αMEM + 100 µM indomethacin, 500 µM isobutylmethylxanthine, 10 µg/mL bovine insulin, and 10^−6^ M dexamethasone, and 10% FBS) was added. Cells were cultured in AIM for up to 14 days with media being changed every 2 days. Parental Scp-1 and PASC-1 cells were differentiated along with Scp-1 p000 and PASC-1 p000 cells under the aforementioned conditions for 21 days. There was no observable difference in adipogenic differentiation (as measured by oil red o staining) between the parental cells and those transduced with the p000 control plasmid (Supplemental Fig. 12D). For circadian expression studies, cells were synchronized with 24 hours of serum starvation. Following serum starvation, the cells were pulse-treated with 100 nM dexamethasone (Sigma) for 2 hours. After dexamethasone treatment the cells were washed with serum free media and then incubated in serum free medium for up to 70 hours. RNA was collected every 6 hours for up to 70 hours.

### Lentiviral Infection

For stable transduction of iBMSCs or ASCs with miR-181a overexpression, miR-181a antagomiR, PER3 shRNA and PPARG shRNA lentiviral vectors HEK 293 T cells were cotransfected with the lentiviral expression vector along with pPACKH1 Packaging Plasmid Mix (System Biosciences) according to the manufacturer provided protocol. Both Scp-1 and PASC-1 cells were then transduced with the lentivirus according to the manufacturer provided protocol. For selection of the iBMSCs or ASCs transduced with miR-181a overexpression (both p000 and p181a vectors) and miR-181a antagomiR (both cmiR and antimiR vectors) RFP FACS was used. For selection of the iBMSCs transduced with PER3 shRNA (both shscram and shPER3 #1 & #2 vectors) and PPARG shRNA (both shscram and shPPARG vectors) GFP FACS was used. For the experiments involving PPARG knockdown the Scp-1 p000 cells were transduced with shscram vector (Scp-1 p000-shscram) and the Scp-1 p181a cells were either transduced with either shscram (Scp-1 p181a-shscram) or shPPARG vectors (Scp-1 p181a-shPPARG). These cells were sorted for the dual RFP/GFP+ cells. All cell lines selected with FACS were analyzed every 3–5 passages to make sure that the RFP and/or GFP+ population was >95% with re-sorting done as needed.

### Plasmids

The following plasmids were used for stable lentiviral transduction of Scp-1 and PASC-1 cells: pPACKH1-GAG, pPACKH1-REV, pVSV-G (Systems Biosciences), pLV-[mir-control], pLV-[mir-181a] (Biosettia), pEZX-AM04-cmiR, pEZX-AM04-anti-miR-181a (Genecopeia), pshPER3 #1 TRCN0000018503, pshPER3 #2 TRCN0000018506, pLKO.1-puro Non-Target shRNA (Sigma), psi-LVRU6GP-shPPARG HSH064513, psi-LVRU6GP-shscram CSHCTR001-LVRU6GH (Genecopeia). For 3′UTR luciferase assays pEZX-MT06-PER3 HmiT021640-MT06 (Genecopeia) was used.

### Luciferase Reporter Assays

3′UTR luciferase reporter assays were carried out using the pEZX-MT06 dual luciferase reporter construct containing both the PER3 3′UTR firefly luciferase reporter as well as a synthetic renilla luciferase reporter under the control of a constitutive promoter (Genecopeia). iBMSC/ASCs were transfected with 1000 ng of the dual PER3 3′UTR/renilla housekeeping plasmid using lipofectamine 2000 (Thermo Fisher Scientific). Cells were harvested and lysed after 24 hr of transfection using the Promega Dual-Luciferase® Reporter Assay System. Luciferase activity was then measured using a GloMax®-Multi Detection System (Promega). Firefly luciferase activity was normalized to the housekeeping renilla luciferase activity.

### miRNA Target Site Prediction

Searches for potential miR-181a targets were done using Targetscan and miRwalk algorithms. Targets were then prioritized based on target relevance to iBMSC adipogenesis, number of binding sites within the 3′UTR, and seed sequence complementarity.

### Oil Red O Staining

iBMSC/ASCs were cultured in AIM for 14 days and then washed in 1X PBS twice followed by fixation in 10% Formalin for 1 hour. Cells were then washed 2X with distilled H_2_O followed by equilibration in 60% isopropanol for 5 minutes. Cells were then stained with Oil Red O in 60% isopropanol for 20 minutes followed by 4 washes with distilled H_2_O. For Oil Red O quantification, 200 µL of 100% isopropanol was added to each well to extract the dye. The absorbance at λ = 500 nm was then measured.

### Quantitative Reverse Transcriptase Polymerase Chain Reaction

Both mRNA and miRNA were isolated using a total RNA isolation kit (Norgen). To determine expression levels of miR-181a, miR-181b, and miR-16 100 ng of total RNA was converted to cDNA using a Taqman Reverse Transcription Kit and Taqman miRNA specific primers (ABI). The miRNA cDNA was then PCR amplified using a Roche Lightcycler II real time PCR machine along with miRNA specific Taqman probes and ABI universal UNG master mix. To determine mRNA expression levels 1000 ng of total RNA was reverse transcribed into cDNA using the Transcriptor Universal cDNA Synthesis Kit (Roche). cDNA was then PCR amplified using a Roche Lightcycler II real time PCR machine along with gene specific PCR primers and LightCycler® 480 SYBR Green I Master Mix (Roche). Primers used are listed below.

**Table 1:** List of primer sequences used in qtRT-PCR analysis.

18S RP	GGAAAGCAGACATTGACCTCAC
18S LP	CCATCCTTTACATCCTTCTGTCTGT
CEBPA RP	TTCACATTGCACAAGGCACT
CEBPA LP	ACGATCAGTCCATCCCAGAG
PPARG RP	CCATTACGGAGAGATCCACG
PPARG LP	AGGCCATTTTGTCAAACGAG
ADPN RP	GAACACCTGTGAGGTCACCC
ADPN LP	CTGTACCCTGCCTGTGGAAT
PLIN RP	AGGTCTTCTGGAAGCATTCG
PLIN LP	CAGTCAACAAAGGCCTCACC
GLUT4 RP	CCCCAATGTTGTACCCAAAC
GLUT4 LP	CTTCCAACAGATAGGCTCCG
FABP4 RP	TGATGATCATGTTAGGTTTGGC
FABP4 LP	TGGAAACTTGTCTCCAGTGAA

### Western Blot

Total protein lysates were prepared from iBMSC pellets using RIPA buffer supplemented with PhosSTOP phosphatase inhibitor and cOmplete protease inhibitor cocktail (Roche). Protein concentration of iBMSC lysates was determined using BCA assay. Western blots were run as described previously^23^. Primary antibodies used are listed below.

**Table 2:** List of primary antibodies used in western blot analysis.

Primary Antibody	Company	Catalog #	Dilution
PPARG	Cell Signaling Technology	2443	1:500
CEBPA	Cell Signaling Technology	2295	1:500
FABP4	Cell Signaling Technology	3544	1:500
PER3	Abcam	ab201940	1:500
GAPDH	Santa Cruz	sc-365062	1:2500
**Secondary Antibody**	**Company**	**Catalog #**	
Mouse anti-rabbit	Cell Signaling Technology	58802	1:2500
Rabbit anti mouse	Cell Signaling Technology	7076	1:2500

## Results

### *miR*-181a is induced during adipogenic BMSC & ASC differentiation

**Figure 1:**
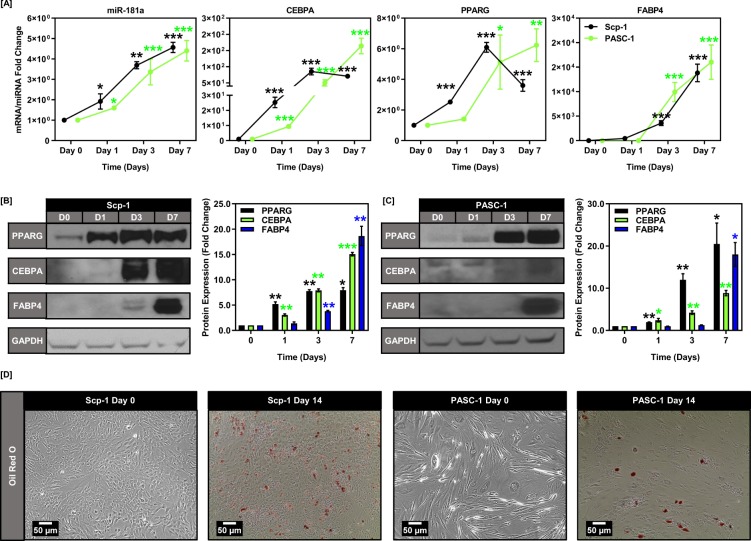
miR-181a is induced during BMSC/ASC adipogenesis. (**A**) Graphs depicting increasing expression of miR-181a during the induction of adipogenesis at days 0, 1, 3, and 7 for Scp-1 and PASC-1 cells. (**B**) Graphs depicting increasing mRNA expression of PPARG, CEBPA, and terminal adipogenesis markers during the induction of adipogenesis at days 0, 1, 3, and 7 for Scp-1 and PASC-1 cells. (**C**) Representative western blot images showing increasing protein expression of PPARG, CEBPA, and FABP4 during the induction of adipogenesis at days 0, 1, 3, and 7 for Scp-1 cells with respective quantifications below (Full blots are shown in Supplemental Fig. 5A). (**D**) Representative western blot images showing increasing protein expression of PPARG, CEBPA, and FABP4 during the induction of adipogenesis at days 0, 1, 3, and 7 for PASC-1 cells with respective quantifications below (Full blots are shown in Supplemental Fig. 5B). (**E**) Micrographs showing Oil red O staining at days 0 and 14 for Scp-1 and PASC-1 cells. Data are representative of 3 independent experiments. * is p ≤ 0.05, ** is p ≤ 0.005, *** is p ≤ 0.0005.

miR-181a has been previously implicated as a potential regulator of adipogenesis^45^. However, the exact mechanistic underpinnings of how miR-181a acts to promote adipogenesis and whether it is involved in BMSC/ASC differentiation are still poorly defined. We first assessed the effects of differentiation on miR-181a expression using two stromal cell models, an immortalized BMSC cell line (Scp-1) and primary adipose derived stromal cells (PASC-1). We found that miR-181a, in conjunction with numerous adipogenic markers, was induced as early as 24 hours into the adipogenic process (Fig. 1A,B). This was further verified by western blot analysis and phenotypically by oil red o staining (Fig. 1C,E). We also found that this correlation was specific to miR-181a and not the co-transcribed miR-181b suggesting that miR-181b was not involved in the adipogenic differentiation process of the Scp-1 or PASC-1 cells (Supplemental Fig. 1).

### Overexpression of miR-181a enhances adipogenic BMSC & ASC differentiation

**Figure 2:**
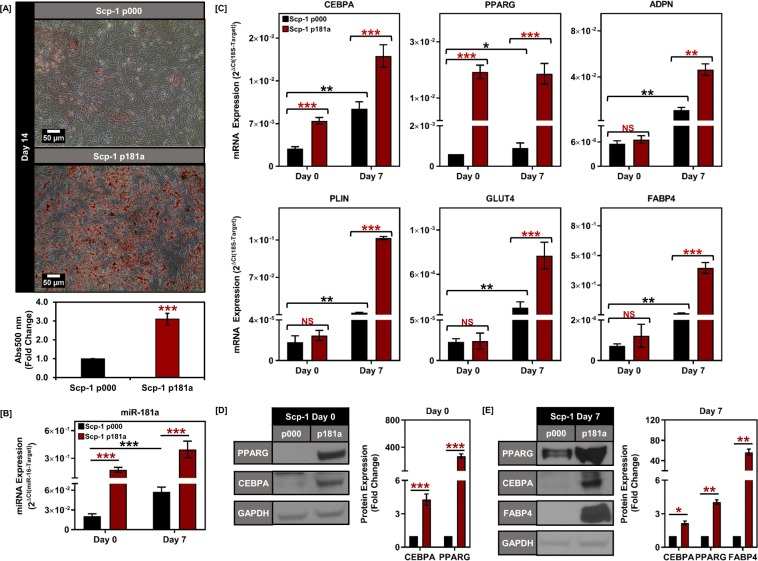
Overexpression of miR-181a in Scp-1 cells increases adipogenesis. (**A**) Oil red O staining for Scp-1 cells overexpressing either scramble vector (p000) or miR-181a (p181a) on day 14 of adipogenic differentiation with representative micrographs (top) and quantification of Oil red O staining for Scp-1 p000 and p181a cells at day 14 (bottom). (**B**) Graph depicting increased expression of miR-181a at days 0 and 7 in Scp-1 p181a vs p000 cells. (**C**) Graphs depicting increased mRNA expression of PPARG and CEBPA at day 0 and increased expression of adipogenesis target genes at day 7 in Scp-1 p181a vs p000 cells. (**D**) Representative western blot images showing increased expression of CEBPA and PPARG at day 0 in Scp-1 p181a vs p000 cells with quantification (right) (Full blots are shown in Supplemental Fig. 6A). (**E**) Representative western blot images showing increased expression of CEBPA, PPARG, and FABP4 at day 7 in Scp-1 p181a vs p000 cells with quantification (right) (Full blots are shown in Supplemental Fig. 6B). Data are representative of 3 independent experiments. * is p ≤ 0.05, ** is p ≤ 0.005, *** is p ≤ 0.0005.

Given the correlation we observed between miR-181a and adipogenesis, we next wanted to determine whether overexpressing miR-181a would promote adipogenesis in the BMSC/ASCs. We stably overexpressed either a control scramble miRNA (p000) or miR-181a (p181a) using a lentiviral vector in both the Scp-1 and PASC-1 cells (Fig. 2B and Supplemental Fig. 2B). Though the miR-181a expressing iBMSC/ASC did not exhibit an increase in visible oil red O staining at Day 0, upon the addition of the adipogenic cocktail there was a robust increase in the adipogenesis in the miR-181a overexpressing iBMSC/ASCs compared to controls at Day 14 (Fig. 2A and Supplemental Fig. 2A). Consistent with these findings, we observed an increase in the levels of PPARG (20-fold in Scp-1 p181a and 5-fold in PASC-1 p181a) and CEBPA (3-fold in Scp-1 p181a and 2-fold in PASC-1 p181a) mRNA and protein levels at Day 0 (Fig. 2C,D and Supplemental Fig. 2C,D, Tables 1 & 2), however, the downstream targets activated by PPARG and CEBPA during adipogenesis were not upregulated at this time point (Fig. 2C and Supplemental Fig. 2C). Interestingly, upon adipogenic stimulus the miR-181a overexpressing BMSC/ASCs retained elevated levels of PPARG and CEBPA as well as increased levels of terminal adipogenic differentiation markers such as ADPN, FABP4, and PLIN through the process of differentiation (Fig. 2C–E and Supplemental Fig. 2C–E). Furthermore, both the BMSC and the ASC cell lines overexpressing miR-181a retained their multipotency and ability to proliferate even after several passages (Supplemental Fig. 12A,B). In addition, the Scp-1 p181a cells did not differ from the Scp-1 p000 cells in terms of proliferation (Supplemental Fig. 12B)

### PER3 is a direct target of miR-181a in Scp-1 and PASC-1 cells

**Figure 3:**
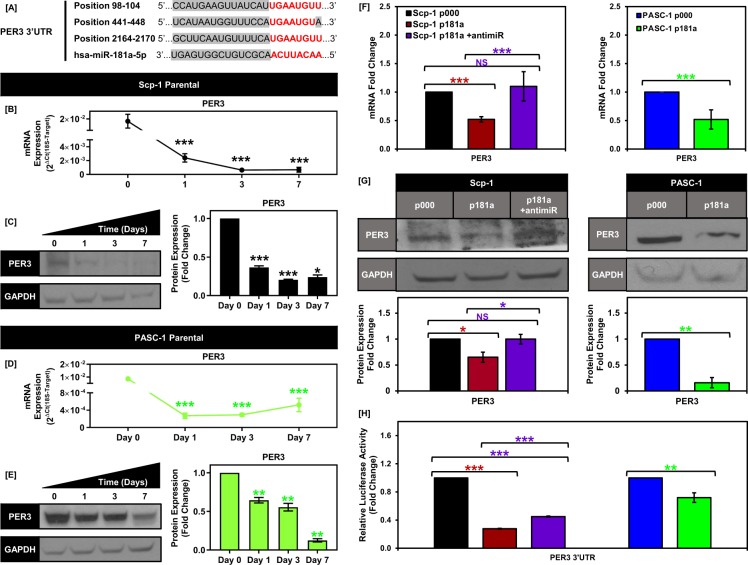
PER3 is a direct target of miR-181a in Scp-1 and PASC-1 cells. (**A**) Diagram depicting the positions of the three predicted, conserved miR-181a binding sites in the 3′UTR of the PER3 gene. (**B**) Graph showing decreasing PER3 mRNA expression for Scp-1 cells. (**C**) Representative western blot images showing decreasing PER3 protein expression (top) following adipogenic induction at days 0, 1, 3, and 7 in Scp-1 cells with quantification (bottom) (Full blots are shown in Supplemental Fig. 6A). (**D**) Graph showing decreasing PER3 mRNA expression for PASC-1 cells. (**E**) Representative western blot images showing decreasing PER3 protein expression (top) following adipogenic induction at days 0, 1, 3, and 7 in PASC-1 cells with quantification (bottom) (Full blots are shown in Supplemental Fig. 7B). (**F**) Graphs showing the PER3 mRNA expression levels for Scp-1 p000, p181a, p181a-antimiR cells (right) and PASC-1 p000 and p181a cells (left). (**G**) Representative westen blot images showing the PER3 protein expression levels for Scp-1 p000, p181a, p181a-antimiR cells and PASC-1 p000 and p181a cells with quantification (below) (Full blots are shown in Supplemental Fig. 7C,D). (**H**) Graph showing the PER3 3′UTR activity levels for Scp-1 p000, p181a, p181a-cmiR, p181a-antimiR cells and PASC-1 p000 and p181a cells. Data are representative of 3 independent experiments. * is p ≤ 0.05, ** is p ≤ 0.005, *** is p ≤ 0.0005.

To further understand the mechanism through which miR-181a was increasing adipogenesis in the iBMSC/ASCs we performed bioinformatics analysis of predicted miR-181a targets using Targetscan and miRwalk. In particular, we focused on negative regulators of PPARG mRNA expression given the robust induction of PPARG observed upon miR-181a expression and found that PER3 was a top predicted target with 3 predicted miR-181a binding sites in its 3′UTR (Fig. 3A). PER3 is a known negative regulator of adipogenesis and we found that PER3 levels decrease during adipogenesis of both the parental Scp-1 and primary PASC-1 cells (Fig. 3B–E). In addition, PER3 mRNA and protein levels were significantly decreased more than 2-fold in the Scp-1 and PASC-1 p181a cells compared to control cells (Fig. 3F,G). Furthermore, the targeted inhibition of miR-181a using a miR-181a specific antagomiR (antimiR-181a) completely abrogated this effect on PER3 (Fig. 3F,G). Next in order to assess whether miR-181a directly targets the PER3 3′UTR we used a 3′UTR luciferase reporter assay. Transfection of the iBMSC/ASC cell lines with the 3′UTR reporter showed a decrease in luciferase activity for the Scp-1 and PASC-1 p181a cells vs controls.

### Knockdown of PER3 phenocopies the effects of miR-181a on BMSC adipogenesis

**Figure 4:**
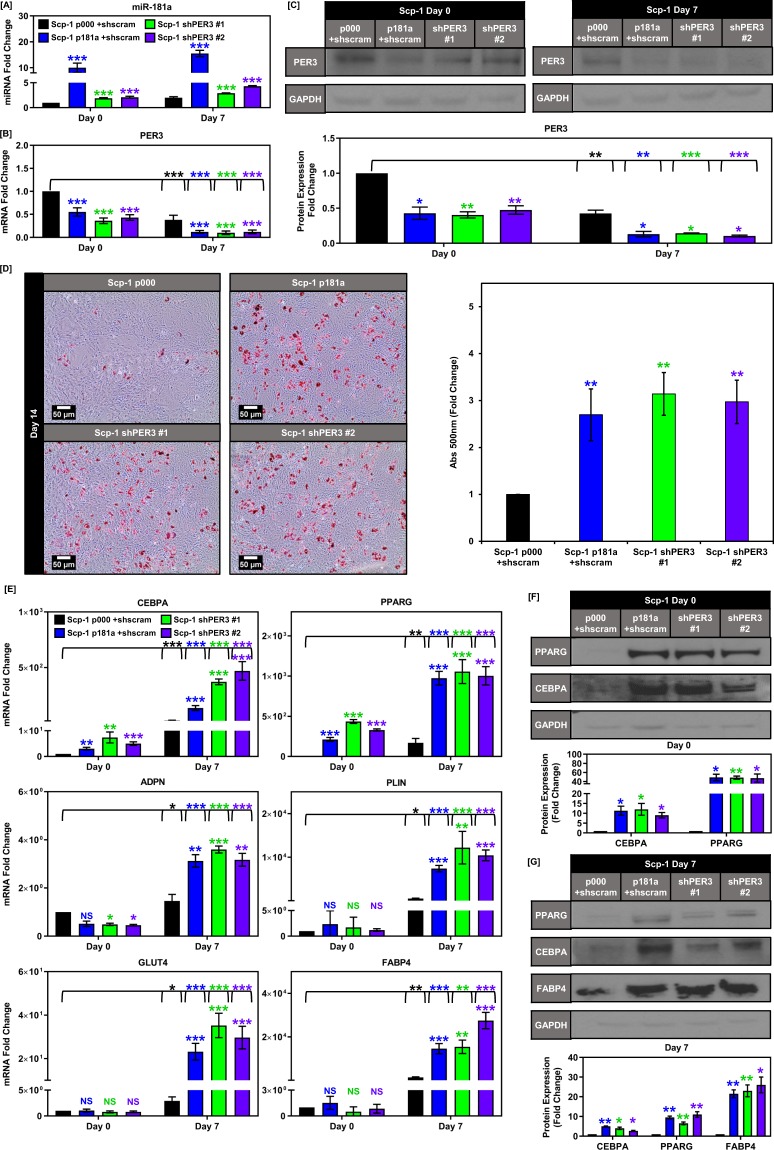
Knockdown of PER3 phenocopies miR-181a overexpression in Scp-1 MSCs. (**A**) Graph showing miR-181a levels at days 0 and 7 of adipogenic differentiation for Scp-1 cells overexpressing either scramble vector (p000) or miR-181a (p181a) or Scp-1 cells expressing PER3 shRNAs (shPER3 #1 & shPER3 #2). (**B**) Graph showing PER3 mRNA levels at days 0 and 7 of adipogenic differentiation in Scp-1 p000, p181a, shPER3 #1, and shPER3 #2 cells. (**C**) Western blot showing PER3 protein levels at days 0 and 7 of adipogenic differentiation in Scp-1 p000, p181a, shPER3 #1, and shPER3 #2 cells (Full blots are shown in Supplemental Fig. 8A,B). (**D**) Oil red O staining images of Scp-1 p000, p181a, shPER3 #1, and shPER3 #2 cells on day 14 of adipogenic differentiation with representative micrographs (top) and quantification of Oil red O staining (bottom). (**E**) Graphs depicting increased mRNA expression of PPARG and CEBPA at day 0 and increased expression of adipogenesis target genes at day 7 in Scp-1 p181a, shPER3 #1, and shPER3 #2 vs p000 cells. (**F**) Representative western blot images showing increased expression of CEBPA and PPARG in Scp-1 p181a, shPER3 #1, and shPER3 #2 vs p000 cells at day 0 with quantification (right) (Full blots are shown in Supplemental Fig. 8B). Representative western blot images showing increased expression of CEBPA, PPARG, and FABP4 at day 7 in Scp-1 p181a, shPER3 #1, and shPER3 #2 vs p000 cells with quantification (right) (Full blots are shown in Supplemental Fig. 8D). Data are representative of 3 independent experiments. * is p ≤ 0.05, ** is p ≤ 0.005, *** is p ≤ 0.0005.

To confirm the causative relationship between miR-181a and PER3, as well as ascertain that PER3 was the miR-181a target responsible for the increase in BMSC adipogenesis, we specifically knocked down PER3 using stable shRNA expression in the bone marrow derived Scp-1 cells. We chose this cell line for our model as it is difficult to perform stable genetic manipulation and long term experimentation of primary BMSC/ASCs without inducing sensecence and/or loss of differentiation potential from serial passaging. We first verified that stable PER3 inhibition lead to similar mRNA and protein downregulation as seen with miR-181a overexpression (Fig. 4B,C). Interestingly, the cells with PER3 knockdown exhibited moderate increases in miR181a expression at baseline (Fig. 4A). Knockdown of PER3 in the Scp-1 cells resulted in a robust increase in adipogenesis comparable to that caused by miR-181a overexpression as measured by oil red o staining as well as increased expression of day 7 adipogenic markers at the mRNA and protein levels (Fig. 4D–G). In addition, the increased basal levels of PPARG and CEBPA found in the iBMSCs overexpressing miR-181a were also present in the shPER3 iBMSCs (Fig. 4E,F). We also confirmed the respective roles of PPARG and miR-181a in the adipogenic phenotype of the Scp-1 p181a cells using an shRNA against PPARG (Supplemental Fig. 3) and a miR-181a antagomiR (Supplemental Fig. 4). We were unable to perform the shPER3, shPPARG, and miR-181a antagomiR experiments in the PASC-1 cells because we could not achieve a robust knockdown due to low transduction efficiency. Taken together these data show that miR-181a targets PER3 which in turn increases baseline expression of PPARG to promote adipogenesis in the iBMSCs.

### Knockdown of endogenous miR-181a inhibits adipogenesis in BMSCs

**Figure 5:**
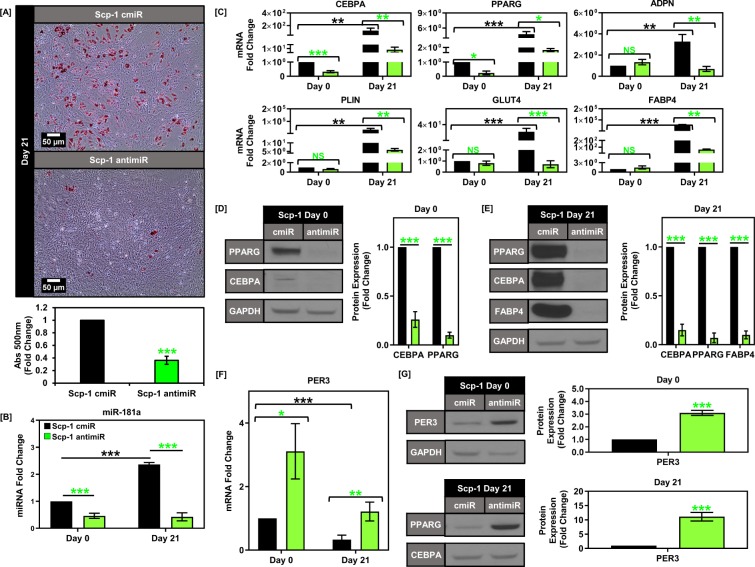
Knockdown of endogenous miR-181a inhibits adipogenesis in BM-MSCs. (**A**) Graph showing miR-181a levels at days 0 and 21 of adipogenic differentiation for Scp-1 cells overexpressing either control vector (cmiR) or miR-181a specific antagomiR (antimiR). (**B**) Oil red O staining images of Scp-1 cmiR and antimiR cells on day 21 of adipogenic differentiation with representative micrographs (top) and quantification of Oil red O staining (bottom). (**C**) Graphs depicting decreased mRNA expression of PPARG and CEBPA at day 0 and decreased expression of adipogenesis target genes at day 21 in Scp-1 antimiR vs cmiR cells. (**D**) Representative western blot images showing decreased expression of CEBPA and PPARG in Scp-1 antimiR vs cmiR cells at day 0 with quantification (right) (Full blots are shown in Supplemental Fig. 9A). (**E**) Representative western blot images showing decreased expression of CEBPA, PPARG, and FABP4 at day 21 in Scp-1 antimiR vs cmiR cells with quantification (right) (Full blots are shown in Supplemental Fig. 9B). (**F**) Graph showing PER3 mRNA levels at days 0 and 21 of adipogenic differentiation in Scp-1 cmiR and antimiR cells. (**G**) Western blot showing PER3 protein levels at days 0 and 21 of adipogenic differentiation in Scp-1 cmiR and antimiR cells (Full blots are shown in Supplemental Fig. 9C,D). Data are representative of 3 independent experiments. * is p ≤ 0.05, ** is p ≤ 0.005, *** is p ≤ 0.0005.

Upon confirming that knockdown of PER3 could phenocopy miR-181a overexpression we next wanted to investigate the effects of endogenous miR-181a regulation of PER3 mediated adipogenic repression. To do this we utilized a miR-181a specific antagomiR and stably overexpressed the antagomiR in the Scp-1 cells followed by induction of adipogenesis. We observed a significant decrease in endogenous expression of miR-181a in the Scp-1 cells expressing the miR-181a antagomiR (approx. 40% of control cells) (Scp-1 antimiR) versus the vector control throughout the differentiation process (Scp-1 cmiR) (Fig. 5A). After 21 days of adipogenic induction, there was a clear decrease in Scp-1 antimiR cell oil red o staining (as low as 35% of control cells) (Fig. 5B). This decrease in adipogenesis of the Scp-1 antimiR cells was further confirmed by qPCR analysis and western blot of adipogenic markers at Day 21 in the Scp-1 cmiR vs antimiR cells (Fig. 5C–E). We confirmed that the adipogenic inhibition effects of the antagomiR were mediated through PER3 by examining PER3 levels in the Scp-1 antimiR cells. We found that there was sustained elevation of PER3 expression at the mRNA and protein levels in the Scp-1 antimiR vs cmiR cells at days 0 and 21 (Fig. 5F,G). Taken together these data show that miR-181a is a key positive regulator of endogenous adipogenic iBMSC differentiation through repression of PER3.

### *miR*-181a modulates PER3 and PPARG circadian rhythm in BMSCs

**Figure 6:**
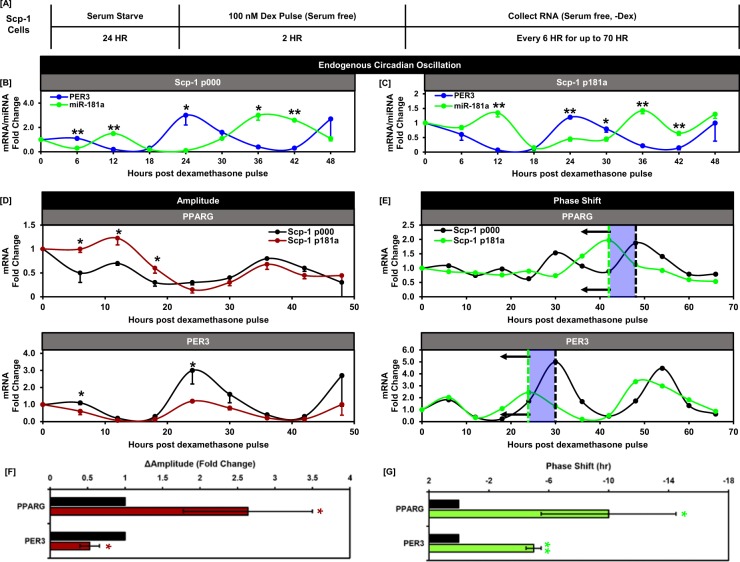
miR-181a and PER3 are regulated in a circadian manner in BM-MSCs. (**A**) Diagram depicting the process of cell synchronization and induction of circadian oscillation using glucocorticoid stimulation. (**B**) Graph depicting the antiphase circadian expression patterns of miR-181a and PER3 in Scp-1 p000 cells. (**C**) Graph depicting the antiphase circadian expression patterns of miR-181a and PER3 in Scp-1 p181a cells. (**D**) Graphs depicting the amplitude changes in circadian expression patterns of PER3 and PPARG in the Scp-1 p181a vs p000 cells. (**E**) Quantification of the amplitude changes in PPARG and PER3 in the Scp-1 p181a vs p000 cells. (**F**) Graphs depicting the phase shift changes in circadian expression patterns of PER3 and PPARG in the Scp-1 p181a vs p000 cells. (**G**) Quantification of the phase-shift changes in PPARG and PER3 in the Scp-1 p181a vs p000 cells. Data are representative of 3 independent experiments. * is p ≤ 0.05, ** is p ≤ 0.005, *** is p ≤ 0.0005.

After observing that miR-181a directly targets PER3 in the iBMSCs we wanted to determine whether miR-181a could regulate circadian circuitry and alter circadian expression of PER3 in a model of circadian oscillation. To investigate circadian regulation of miR-181a and PER3 we utilized an established *in vitro* model of circadian oscillation where the iBMSCs were serum starved for 24 hours (to arrest cell proliferation) followed by a 2 hour dexamethasone pulse (to induce oscillation of the circadian feedback loop within the iBMSCs) and serial collection every 6 hours for up to 70 hours under serum free conditions^46,47^ (Fig. 6A). miR-181a exhibited an oscillatory pattern of expression during the time course indicating that it is regulated in a circadian fashion *in vitro* (Fig. 6B). Interestingly, PER3 exhibited oscillatory expression that was antiphase with both miR-181a and PPARG (Fig. 6B,C). In addition, in the Scp-1 p181a cells both the starting and overall amplitude of PER3 oscillation were reduced compared to the Scp-1 p000 cells (Fig. 6B,C) (Δamplitude approximately 0.5 vs 1 in the Scp-1 p181a vs p000 cells) indicating sustained upregulation of miR-181a influences the amplitude of this circadian target. Furthermore, increased expression of miR-181a also increased the amplitude of PPARG circadian expression (approximately 2.5 fold in the Scp-1 p181a vs p000 cells) providing further evidence for a miR-181a-PER3-PPARG regulatory axis in circadian rhythm. Intriguingly, by extending the circadian time course out past 48 hours we also observed a phase delay in both PPARG and PER3 oscillation by 70 hours in the p181a cells (Fig. 6F,G). Taken together these data establish for the first time that miR-181a is regulated in a circadian manner and can modulate the oscillation properties of circadian rhythm components.

## Discussion

BMSCs and ASCs are the primary source for generating new adipocytes in bone marrow and white adipose tissue respectively. The development of adipocytes from BMSC/ASCs has been an area of intense study for many years as adipocyte related pathologies such as bone marrow adiposity and obesity have developed into major healthcare problems. Thus, elucidating the molecular mechanisms that are involved in BMSC/ASC adipogenesis is essential to understanding adipocyte related pathologies as well as developing therapeutic interventions^48^.

Previous research done both by our lab and others has implicated miR-181a as a key regulator that can promote either stem-like properties or differentiation depending on the biological context. In normal cell biology miR-181a plays an important role as an established driver of differentiation. For example, it has been reported as an important driver of differentiation in immune B-cells and megakaryocytes as well as myoblasts^41,43,49^. Thus, we sought to explore whether miR-181a would promote differentiation in BMSC/ASCs where the function of miR-181a is less well-defined. In particular, it has recently been suggested that the miR-181 family could potentially be regulated in a circadian manner^50,51^. Given the important role of circadian regulation in BMSC/ASC adipogenesis^29–33^, we wanted to assess whether miR-181a could affect circadian clock components to influence adipogenesis.

Initially, we investigated how endogenous miR-181a levels correlated with different types of multipotent stromal cell differentiation in both immortalized (Scp-1) BMSCs and primary (PASC-1) ASCs. We hypothesized that positive or negative correlation between miR-181a levels and differentiation could be indicative of a functional role for miR-181a. Our results showed that miR-181a exhibited a strong positive correlation with adipogenesis in both cell lines and that the correlation was specific to miR-181a and not the co-transcribed miR-181b. Overexpression of miR-181a in Scp-1 and PASC-1 cells resulted in a robust increase in adipogenesis as evidenced by the increase in adipogenic markers and lipid accumulation. A key observation from these experiments was that there was a sizable increase in the levels of PPARG mRNA and protein in the Scp-1 and PASC-1 p181a cells at baseline without the addition of any adipogenic inducers. Transcription of PPARG is tightly regulated in BMSC/ASCs through transcription factors and co-factors that act as repressors at the PPARG promoter^52^. Typically, PPARG transcription levels in BMSC/ASCs remain relatively low in the absence of adipogenic cues and upon adipogenic induction the PPARG promoter then undergoes a stepwise process involving both epigenetic modifications and the exchange of transcriptional repressors for activators to become fully active. Intriguingly, even though we observed an increase in PPARG and CEBPA levels at Day 0 in the miR-181a overexpressing cells (relative to control iBMSC/ASCs) prior to adipogenic induction, their downstream targets remained unaffected. However, following the addition of adipogenic cocktail to these cells there was a significant increase in expression of these target genes between the control and miR-181a overexpressing iBMSC/ASCs, suggesting that the increase in miR-181a expression was ‘priming’ these cells and transitioning the PPARG gene from a basal state of low transcriptional activity to a higher level of transcriptional activity prior to adipogenic differentiation. Furthermore, this increase in PPARG transcriptional activity was maintained even after adipogenic induction. Taken together these data pointed toward a decrease in a transcriptional repressor upstream of PPARG as epigenetic rearrangement of chromatin structure that allows for complete activation of PPARG requires stimulation of cAMP signaling which only happens after addition of the adipogenic cocktail *in vitro*.

Using bioinformatic analysis we examined known transcriptional repressors of PPARG and identified period circadian regulator 3 (PER3) as a promising target for miR-181a. PER3 is the least studied of the three PER genes has 3 conserved miR-181a binding sites in its 3′UTR (Fig. 4A) and an established role as a negative regulator of PPARG. We found that PER3 was decreased at the mRNA and protein level in iBMSC/ASCs that overexpressed miR-181a. Furthermore, our 3′UTR data showed that miR-181a directly targets PER3 in Scp-1 & PASC-1 p181a cells and that the 3′UTR activity could be partially rescued by addition of miR-181a antagomiR (Fig. 4H). We also observed that endogenous regulation of PER3 by miR-181a is critical for adipogenesis in the Scp-1 cells (Fig. 5). Circadian regulation of PPARG is well established with reports showing that core clock components such as PER can regulate PPARG in a circadian fashion^30–32^. In addition, other studies have shown that PPARG itself can act to regulate the core clock components in the circadian control of processes such as blood pressure^29,53^. Of the 3 PER genes, PER2 and PER3 have been shown in mouse knockout models to inhibit PPARG either through direct binding interaction or transcriptional regulation^31,32^. These findings have been expanded in a recent report focused on ASCs that details how PER3 acts as a central component to repress adipogenesis as well as a principal component of the peripheral clock in ASCs *in vivo*^33^. However, the regulation of PER genes, including PER3, by miRNAs has not been well-characterized. Here we report a new mechanism by which one miRNA, miR-181a, can promote adipogenesis by directly targeting PER3, a component of the circadian machinery.

Our results place miR-181a at an interesting nexus of both the adipogenesis and circadian rhythm of BMSC/ASCs. By targeting PER3 at baseline miR-181a acts to “prime” the BMSC/ASCs to undergo adipogenesis by elevating basal levels of PPARG. The phenomenon of “priming” multipotent BMSCs to favor differentiation into a particular lineage is well documented^54–56^. There is considerable heterogeneity within multipotent BMSCs *in vivo* with several subpopulations that are primed for differentiation into a particular lineage or lineages. Both the priming and commitment of BMSCs to a particular lineage is influenced by both cell intrinsic factors (i.e. the levels of early commitment transcription factors) as well as cell extrinsic factors (i.e. paracrine and endocrine signaling). In our models the stable overexpression of miR-181a increased PPARG levels without forcing differentiation suggesting that the cells are primed for adipogenic differentiation with the addition of the proper extrinsic cues. These results have interesting implications for BMSC/ASC related pathologies. It could be hypothesized that in the bone marrow compartment BMSCs with a high level of miR-181a and therefore PPARG would be predisposed toward adipogenesis and could contribute to bone formation imbalance and impair hematopoiesis. In the adipose compartment miR-181a high ASCs that are primed for adipogenesis could contribute to increased adipose mass which in turn contributes to obesity and its related diseases. In addition, by showing that miR-181a repression of PER3 drives adipogenesis in MSCs derived from two different compartments (bone marrow and visceral adipose tissue) our data support the idea of PER3 mediated repression of adipogenesis as a potential clinically relevant mechanism of adipogenic regulation in MSCs.

With respect to circadian rhythm, our results indicate that by targeting PER3 in BMSCs miR-181a not only affects PER3 mediated repression of adipogenesis but also alters the circadian oscillation of PER3 as well as downstream targets under its control such as PPARG. We observed that in control BMSCs both miR-181a and PPARG appear to be anti-phase to PER3 in terms of their oscillation expression. PPARG is a direct target of PER3 repression and thus expected to be anti-phase to PER3. However, the transcriptional regulation of miR-181a is not well defined, particularly in the context of circadian rhythm, apart from the fact that miR-181 family members have been shown to oscillate in a circadian fashion. One possible explanation is that like PPARG, miR-181a is under the regulation of the circadian machinery opposite to PER3. Further studies will be needed to more clearly determine where miR-181a acts within the circadian network. Stably increasing miR-181a expression in the iBMSCs caused both changes in amplitude and phase of PER3 and PPARG oscillation. The changes in amplitude (increased for PPARG and decreased for PER3) can be attributed to the decrease of PER3 levels as a result of miR-181a targeting. Interestingly, both PER3 and PPARG experienced a phase delay within 72 hours of oscillation induction. This can be explained by the fact that the stably increased expression of miR-181a acts as brake on PER3 accumulation thereby delaying the oscillation cycle of PER3 and by extension PPARG. Disruption of circadian cycles in humans, including phase-delay, have a direct correlation with increased BMI, obesity, and metabolic syndrome^57–61^. Our data suggest that miR-181a may act as a mediator of phase-delay in the peripheral clocks of BMSCs and thus contribute to the metabolic complications associated with circadian disruption. miR-181a has already been positively correlated with diabetic inflammation in mice and humans so it could be postulated that chronic stimuli such as inflammation could lead to prolonged increases in miR-181a expression in MSCs necessary to cause circadian dysregulation^62^.

There still remain several unanswered questions which require further study. The most important question is whether the miR-181a-PER3-PPARG mechanism occurs in BMSC/ASCs *in vivo* and whether it is a generalized phenomenon or occurs only in specific compartments. In addition, the signaling factors and pathways that increase miR-181a expression in BMSCs and ASCs both *in vitro* and *in vivo* will need to be elucidated. Another important question is whether certain disease states (i.e. obesity, inflammation) might increase BMSC/ASC miR-181a expression in a sustained manner. Our results show that miR-181a can be regulated in a circadian fashion and that this expression is in synchrony with PPARG expression and antiphase to PER3 expression. Further studies will be needed to determine whether this mechanism also occurs in bone marrow and adipose derived stromal cells *in vivo*. However, there is some evidence in the literature to suggest a functional *in vivo* circadian relationship between PPARG and miR-181a. Yang *et al*. examined the circadian expression of nuclear receptors *in vivo* and found that PPARG and GCNF (which contains miR-181a as an intronic miRNA) exhibited similar patterns of circadian expression^63^. In addition to the circadian behavior of miR-181a, we found that overexpressing miR-181a also altered circadian expression of PER3 and PPARG by decreasing and increasing the amplitude of expression, respectively. The circadian regulation of miR-181a demonstrated in our BMSC/ASC models provides an initial lead to suggest that miR-181a has a role in circadian biology and its associated diseases. Our data raise interesting questions about whether miR-181a can act on other targets that are part of the circadian machinery and whether that would have the same effect on BMSC/ASC differentiation. Taken together our results show a previously unknown role for miR-181a to promote BMSC/ASC differentiation by targeting a circadian factor and implicate miR-181a as a novel component of the BMSC circadian regulatory network that can contribute to circadian dysregulation in BMSC and potentially ASCs.

## Electronic supplementary material

Supplemental Data
