# Supplementary material for: miR-181a modulates circadian rhythm in immortalized bone marrow and adipose derived stromal cells and promotes differentiation through the regulation of PER3
Source: Sci Rep. 2019 Jan 22;9:307. doi: 10.1038/s41598-018-36425-w (PMC6343011; doi:10.1038/s41598-018-36425-w)
Supplement: Supplementary file 1 — Supplemental Data [file 41598_2018_36425_MOESM1_ESM.pdf]

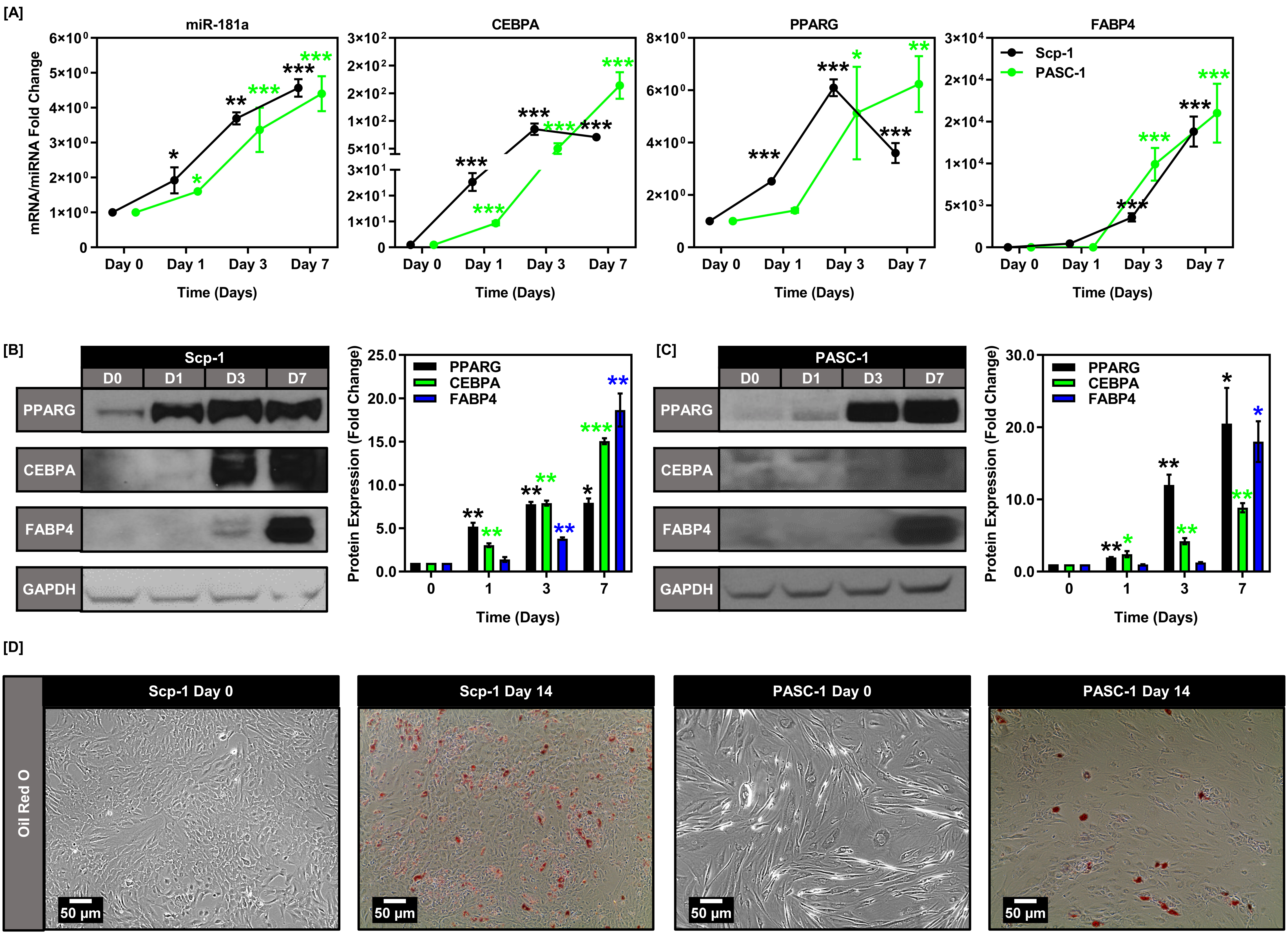
[A]
miR-181a
CEBPA
PPARG
FABP4
mRNA/miRNA Fold Change
Scp-1
PASC-1
Day 0
Day 1
Day 3
Day 7
Time (Days)
[B]
Scp-1
D0
D1
D3
D7
PPARG
CEBPA
FABP4
GAPDH
Protein Expression (Fold Change)
Time (Days)
[C]
PASC-1
[D]
Scp-1 Day 0
Scp-1 Day 14
PASC-1 Day 0
PASC-1 Day 14
Oil Red O
50 μm

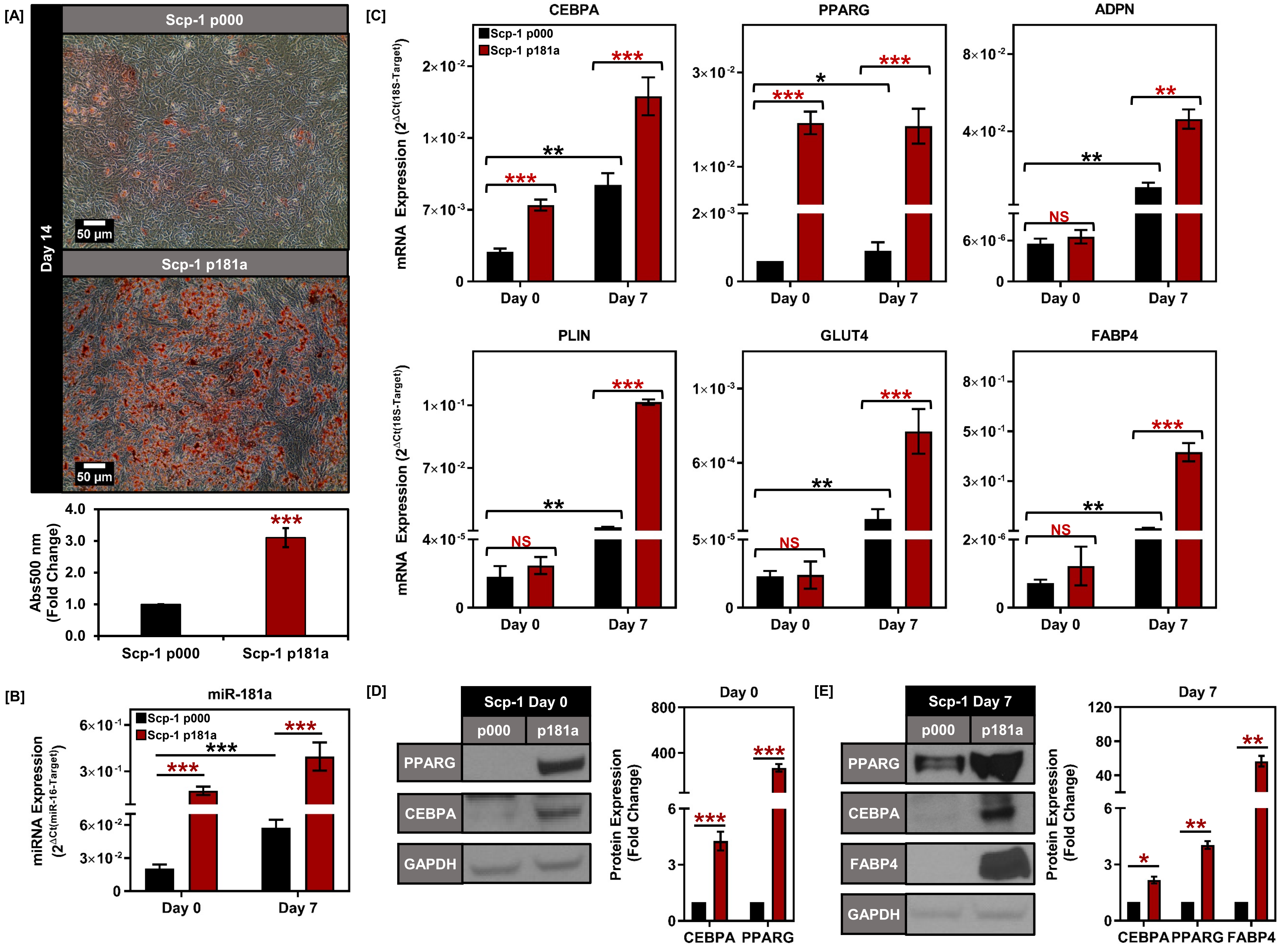

[A]
Scp-1 p000
Day 14
50 µm
Scp-1 p181a
50 µm
Abs500 nm (Fold Change)
4.0
3.0
2.0
1.0
0.0
***
Scp-1 p000
Scp-1 p181a
[B]
miR-181a
miRNA Expression
Scp-1 p000
Scp-1 p181a
6×10-1
3×10-1
6×10-2
3×10-2
0
***
***
***
Day 0
Day 7
[C]
CEBPA
PPARG
ADPN
mRNA Expression
Scp-1 p000
Scp-1 p181a
2×10-2
1×10-2
7×10-3
0
3×10-2
1×10-2
2×10-3
8×10-2
4×10-2
6×10-6
NS
*
**
***
Day 0
Day 7
PLIN
GLUT4
FABP4
1×10-1
7×10-2
4×10-5
1×10-3
6×10-4
5×10-5
8×10-1
5×10-1
3×10-1
2×10-6
[D]
Scp-1 Day 0
p000
p181a
PPARG
CEBPA
GAPDH
Day 0
Protein Expression (Fold Change)
800
400
6
3
0
CEBPA
PPARG
[E]
Scp-1 Day 7
p000
p181a
PPARG
CEBPA
FABP4
GAPDH
Day 7
120
80
40
6
3
0
CEBPA
PPARG
FABP4

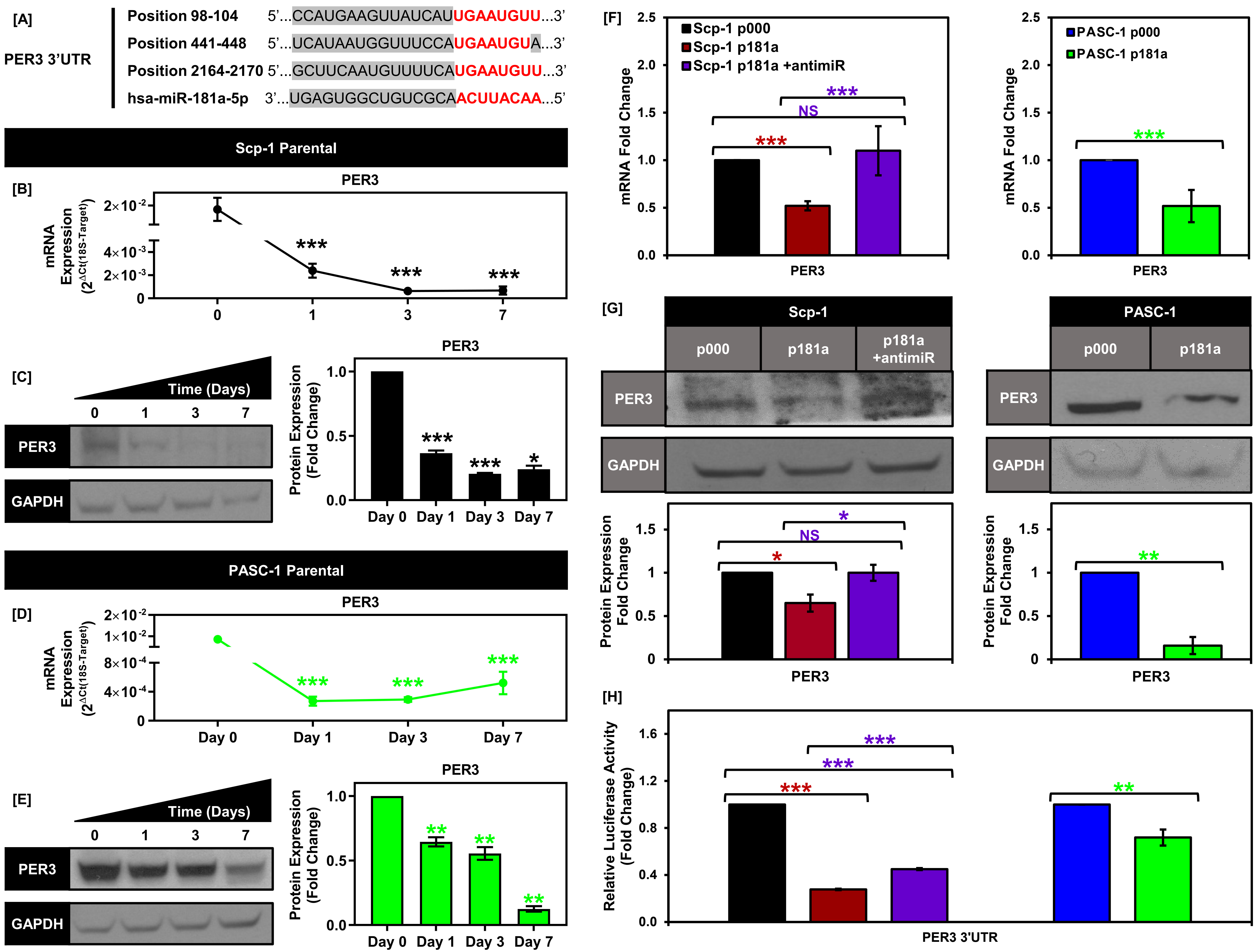
[A]
PER3 3'UTR
Position 98-104 5'...CCAUGAAGUUAUCAUUGAAUGUU...3'
Position 441-448 5'...UCAUAAUGGUUUCCAUGAAUGUA...3'
Position 2164-2170 5'...GCUUCAAUGUUUUCAUGAAUGUU...3'
hsa-miR-181a-5p 3'...UGAGUGGCUGUCGCAACUUACAA...5'
Scp-1 Parental
[B]
PER3
mRNA Expression (2^ΔCt(18S-Target))
0 1 3 7
***
[C]
Time (Days)
0 1 3 7
PER3
GAPDH
Protein Expression (Fold Change)
Day 0 Day 1 Day 3 Day 7
PASC-1 Parental
[D]
Day 0 Day 1 Day 3 Day 7
[E]
[F]
Scp-1 p000
Scp-1 p181a
Scp-1 p181a +antimiR
PASC-1 p000
PASC-1 p181a
mRNA Fold Change
NS
[G]
Scp-1
PASC-1
p000
p181a
p181a +antimiR
Protein Expression Fold Change
[H]
Relative Luciferase Activity (Fold Change)
PER3 3'UTR

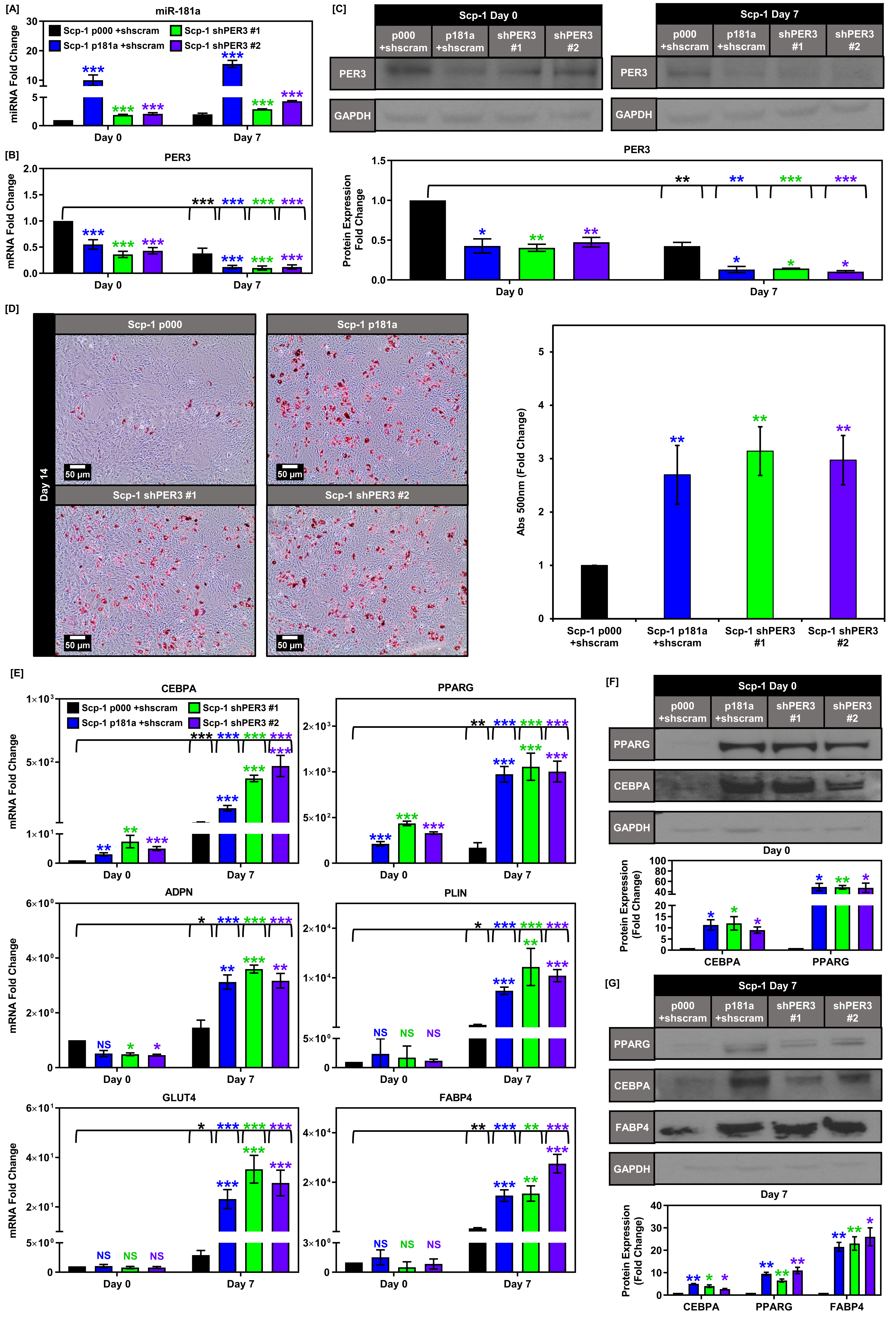

[A]
miR-181a
miRNA Fold Change
Scp-1 p000 +shscram
Scp-1 p181a +shscram
Scp-1 shPER3 #1
Scp-1 shPER3 #2
Day 0
Day 7
[B]
PER3
mRNA Fold Change
Day 0
Day 7
[C]
Scp-1 Day 0
Scp-1 Day 7
p000 +shscram
p181a +shscram
shPER3 #1
shPER3 #2
PER3
GAPDH
PER3
Protein Expression Fold Change
Day 0
Day 7
[D]
Day 14
Scp-1 p000
Scp-1 p181a
Scp-1 shPER3 #1
Scp-1 shPER3 #2
50 µm
Abs 500nm (Fold Change)
Scp-1 p000 +shscram
Scp-1 p181a +shscram
Scp-1 shPER3 #1
Scp-1 shPER3 #2
[E]
CEBPA
PPARG
ADPN
PLIN
GLUT4
FABP4
mRNA Fold Change
Day 0
Day 7
NS
[F]
Scp-1 Day 0
p000 +shscram
p181a +shscram
shPER3 #1
shPER3 #2
PPARG
CEBPA
GAPDH
Day 0
Protein Expression (Fold Change)
CEBPA
PPARG
[G]
Scp-1 Day 7
p000 +shscram
p181a +shscram
shPER3 #1
shPER3 #2
PPARG
CEBPA
FABP4
GAPDH
Day 7
Protein Expression (Fold Change)
CEBPA
PPARG
FABP4

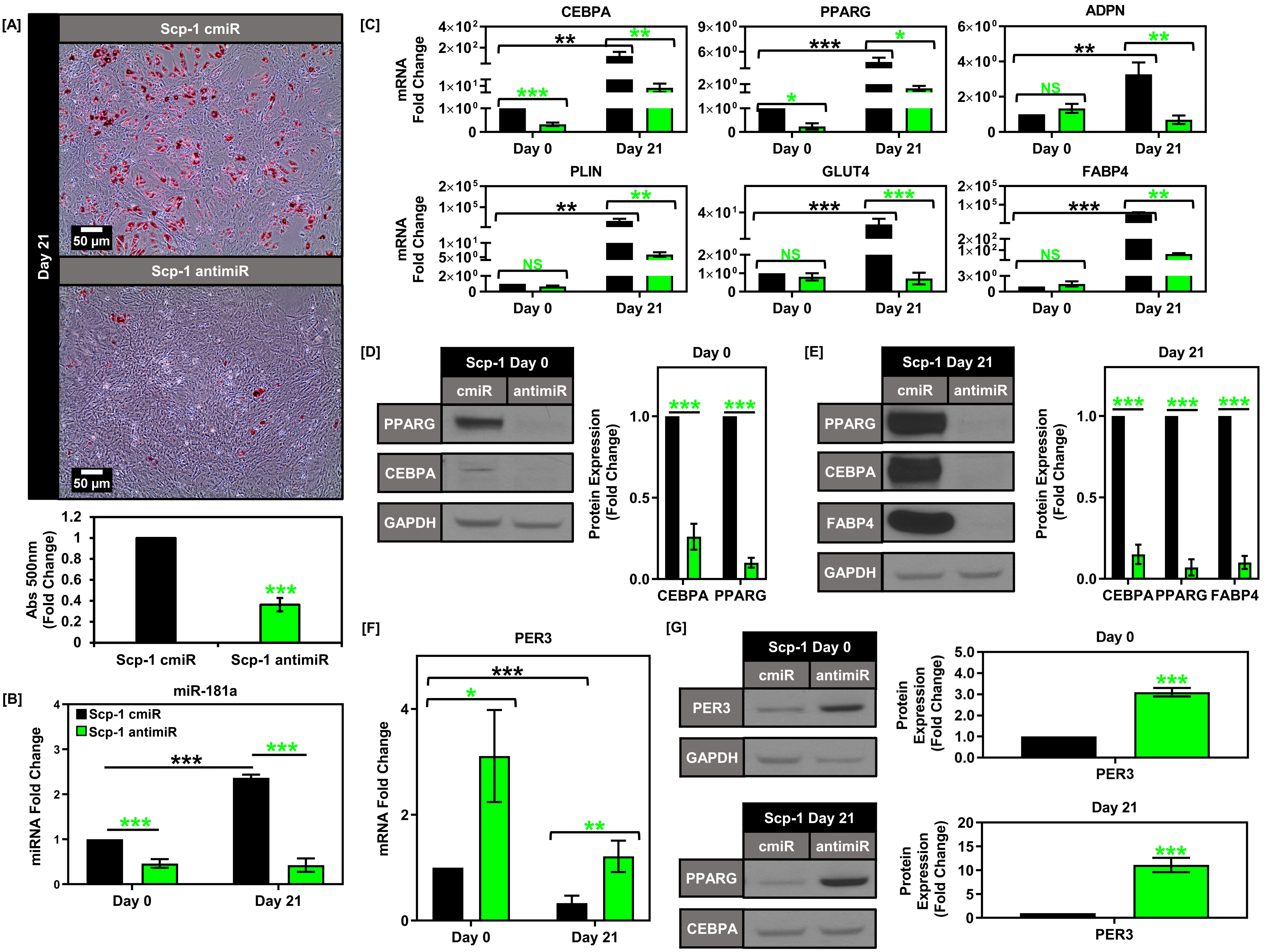
[A]
Day 21
Scp-1 cmiR
Scp-1 antimiR
50 µm
Abs 500nm (Fold Change)
Scp-1 cmiR
Scp-1 antimiR
***
[B]
miR-181a
miRNA Fold Change
Scp-1 cmiR
Scp-1 antimiR
Day 0
Day 21
[C]
mRNA Fold Change
CEBPA
PPARG
ADPN
PLIN
GLUT4
FABP4
NS
Day 0
Day 21
[D]
Scp-1 Day 0
cmiR
antimiR
PPARG
CEBPA
GAPDH
Day 0
Protein Expression (Fold Change)
[E]
Scp-1 Day 21
cmiR
antimiR
PPARG
CEBPA
FABP4
GAPDH
Day 21
Protein Expression (Fold Change)
[F]
PER3
mRNA Fold Change
Day 0
Day 21
[G]
Scp-1 Day 0
cmiR
antimiR
PER3
GAPDH
Scp-1 Day 21
PPARG
CEBPA
Day 0
Day 21
Protein Expression (Fold Change)
PER3

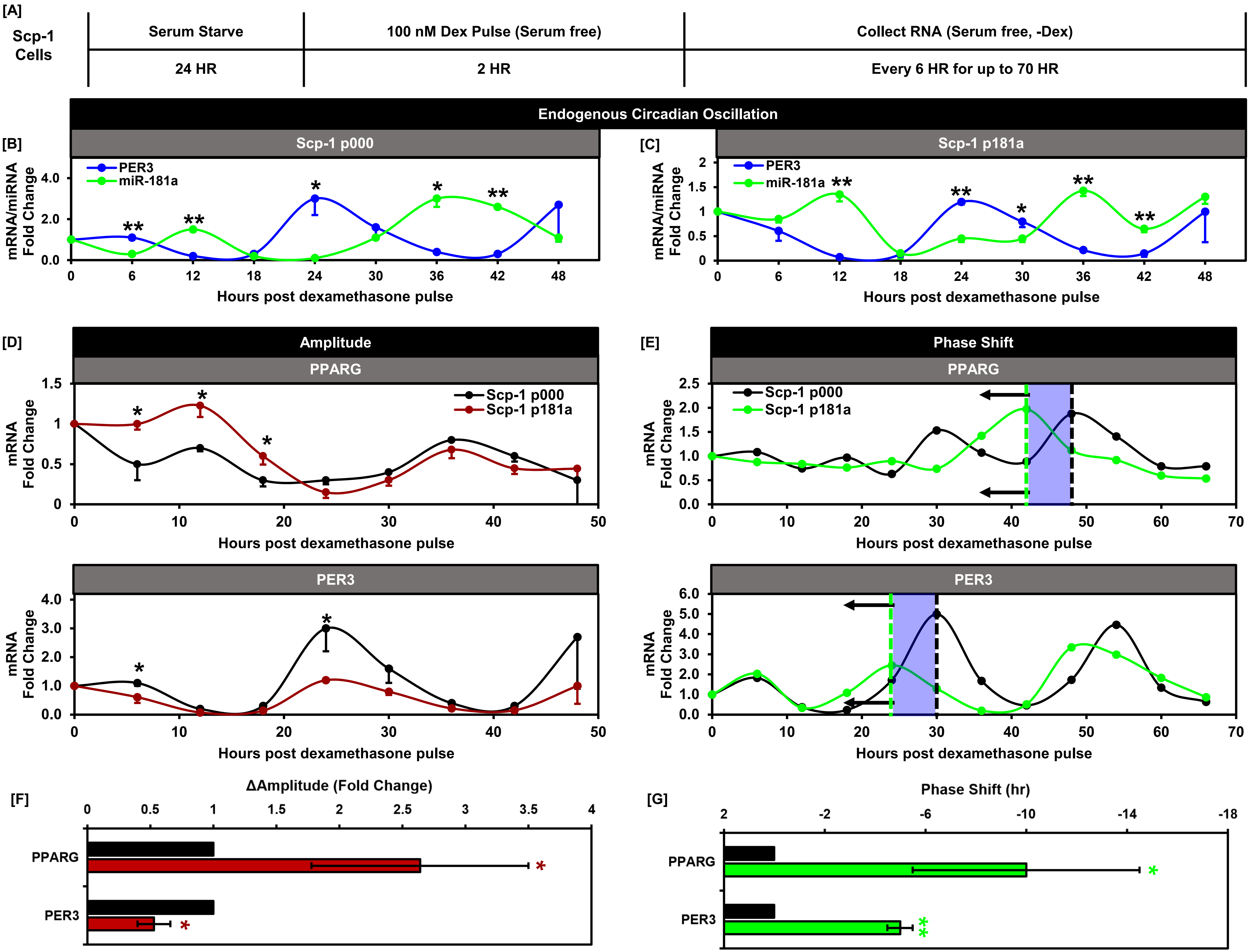

[A]
Scp-1 Cells
Serum Starve
24 HR
100 nM Dex Pulse (Serum free)
2 HR
Collect RNA (Serum free, -Dex)
Every 6 HR for up to 70 HR
Endogenous Circadian Oscillation
[B]
Scp-1 p000
PER3
miR-181a
mRNA/miRNA Fold Change
Hours post dexamethasone pulse
[C]
Scp-1 p181a
PER3
miR-181a
mRNA/miRNA Fold Change
Hours post dexamethasone pulse
[D]
Amplitude
PPARG
Scp-1 p000
Scp-1 p181a
mRNA Fold Change
Hours post dexamethasone pulse
PER3
mRNA Fold Change
Hours post dexamethasone pulse
[E]
Phase Shift
PPARG
Scp-1 p000
Scp-1 p181a
mRNA Fold Change
Hours post dexamethasone pulse
PER3
mRNA Fold Change
Hours post dexamethasone pulse
[F]
ΔAmplitude (Fold Change)
PPARG
PER3
[G]
Phase Shift (hr)
PPARG
PER3

Supplemental Figure 1

[A]

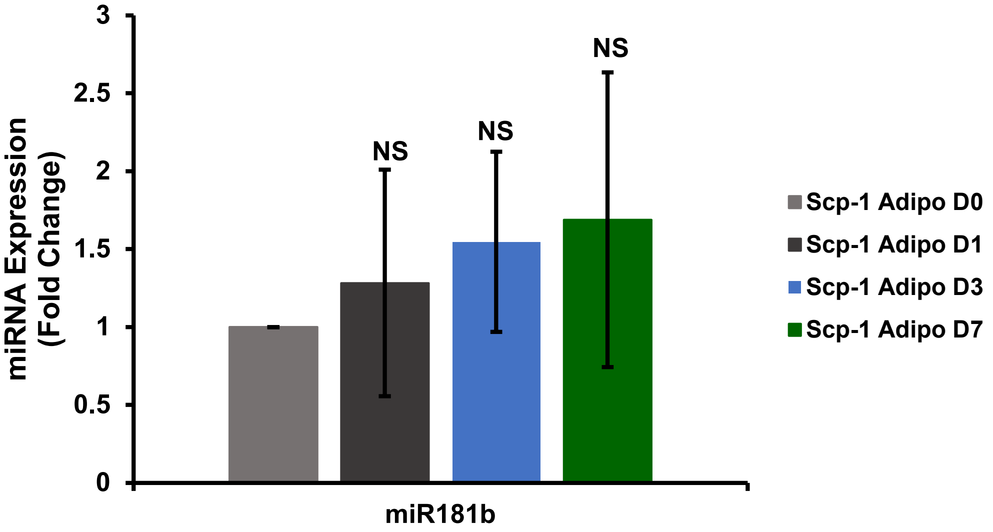

[B]

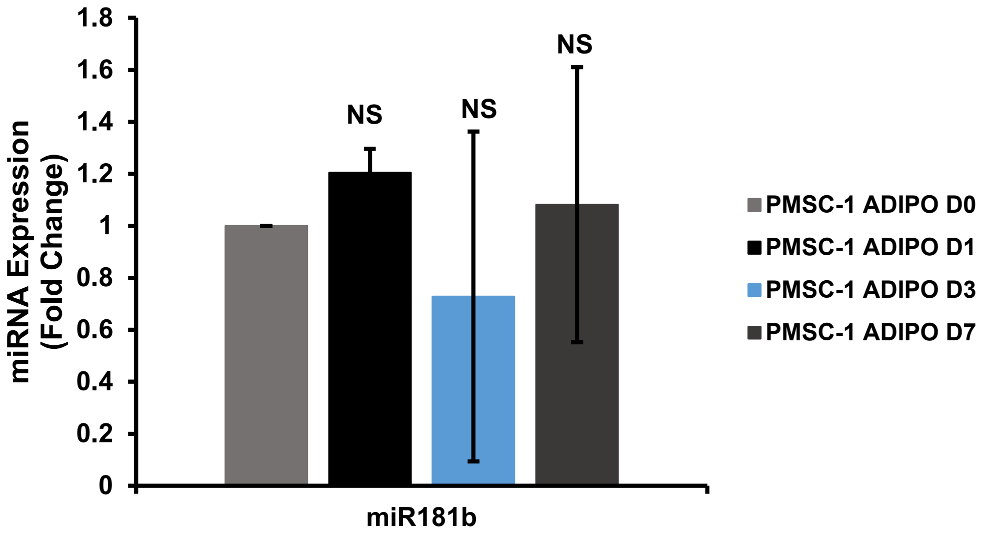

Supplemental Figure 2

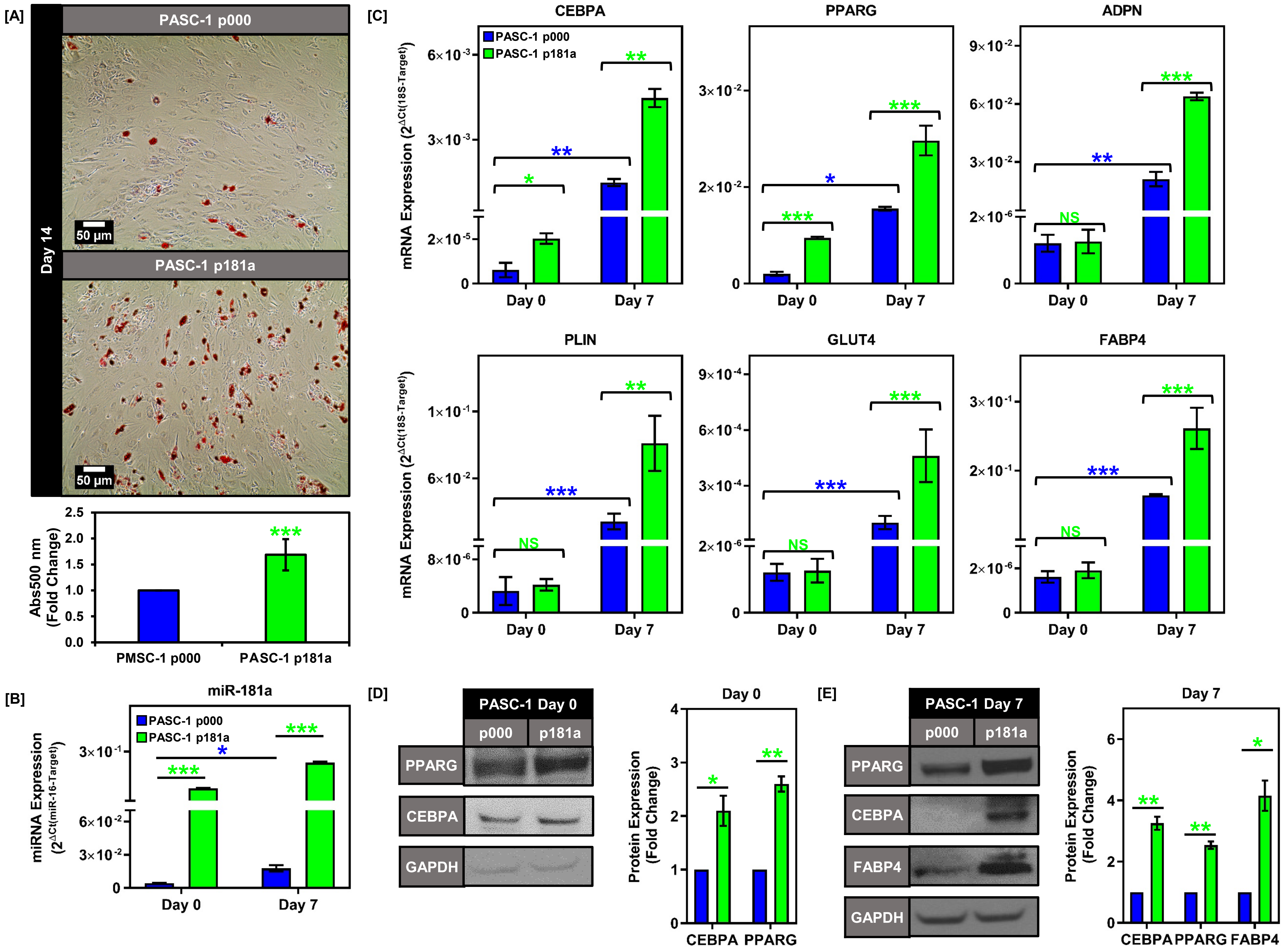

Supplemental Figure 3

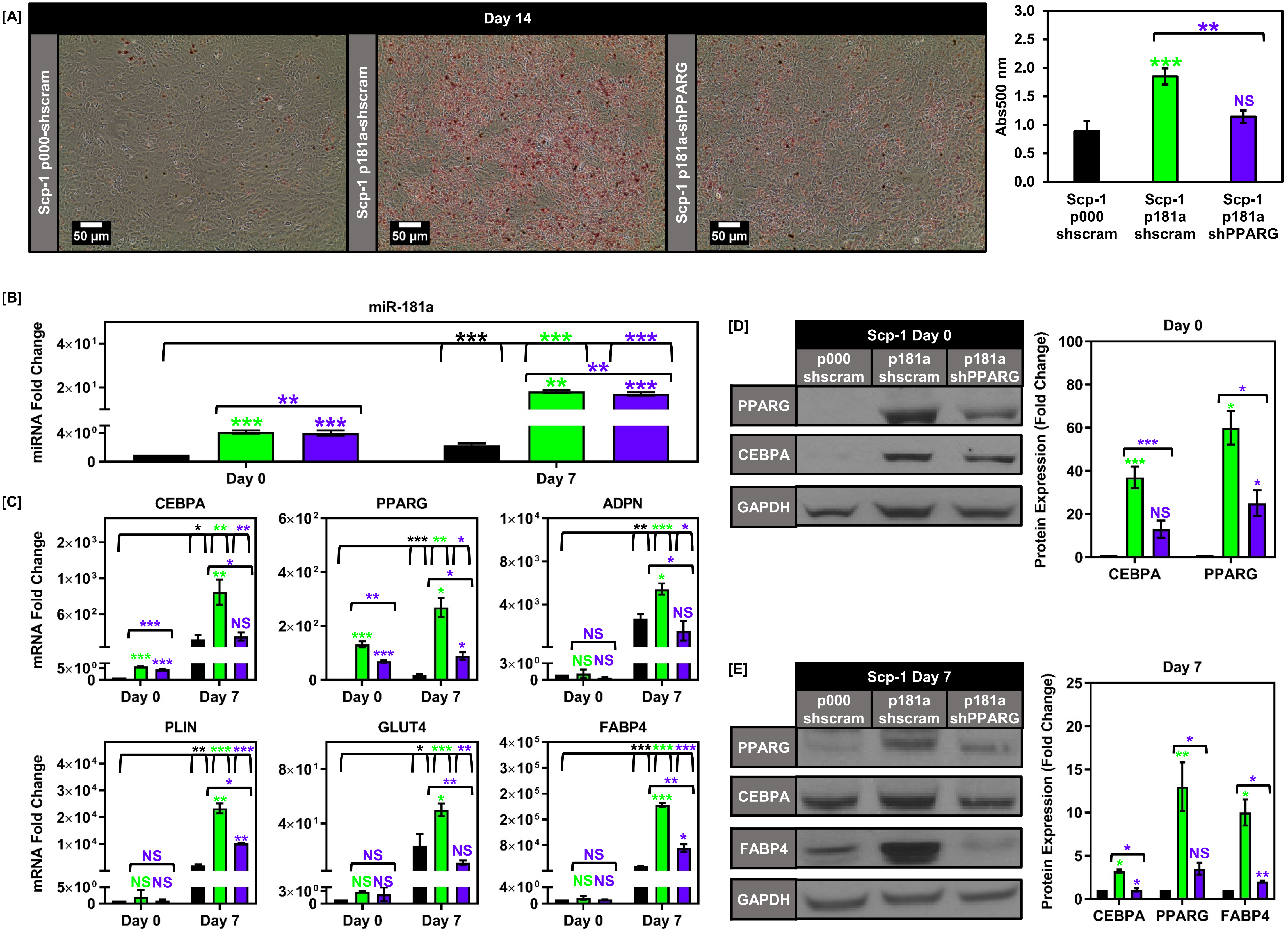

Supplemental Figure 4

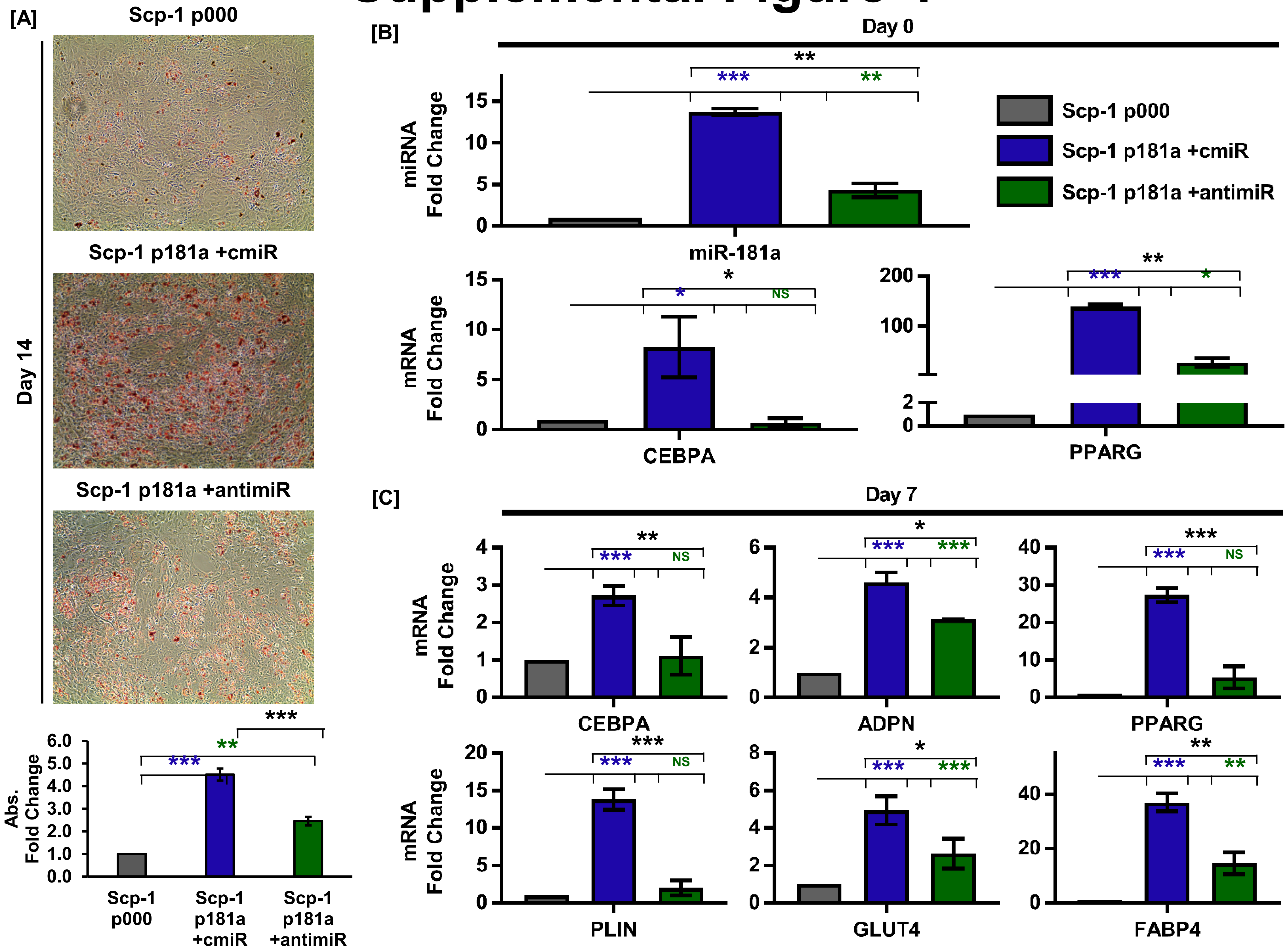

Supplemental Figure 5

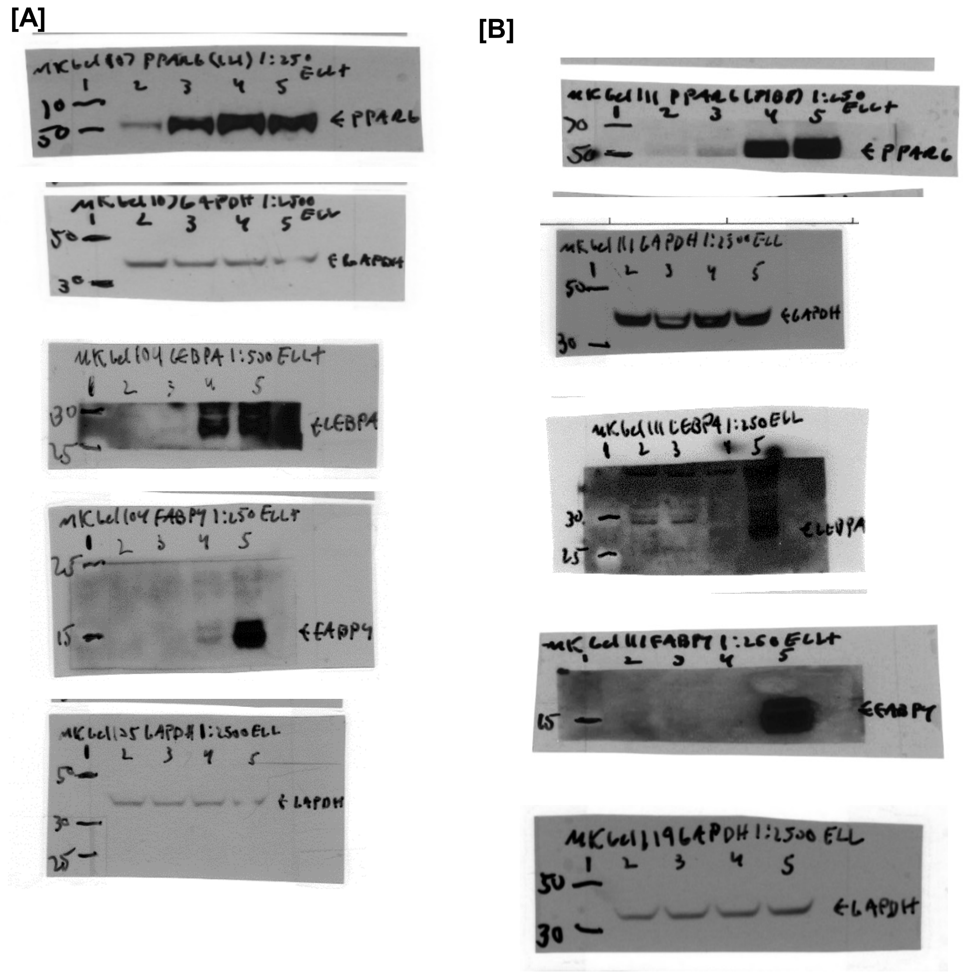

Supp. Fig. 5A (All Gels)	
**Lane 1**	Ladder
**Lane 2**	Scp-1 Adipo Day 0
**Lane 3**	Scp-1 Adipo Day 1
**Lane 4**	Scp-1 Adipo Day 3
**Lane 5**	Scp-1 Adipo Day 7

Supp. Fig. 5B (All Gels)	
**Lane 1**	Ladder
**Lane 2**	PASC-1 Adipo Day 0
**Lane 3**	PASC-1 Adipo Day 1
**Lane 4**	PASC-1 Adipo Day 3
**Lane 5**	PASC-1 Adipo Day 7

Supplemental Figure 6

[A]

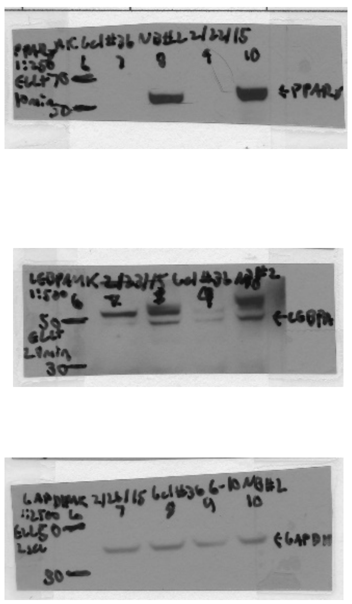

[B]

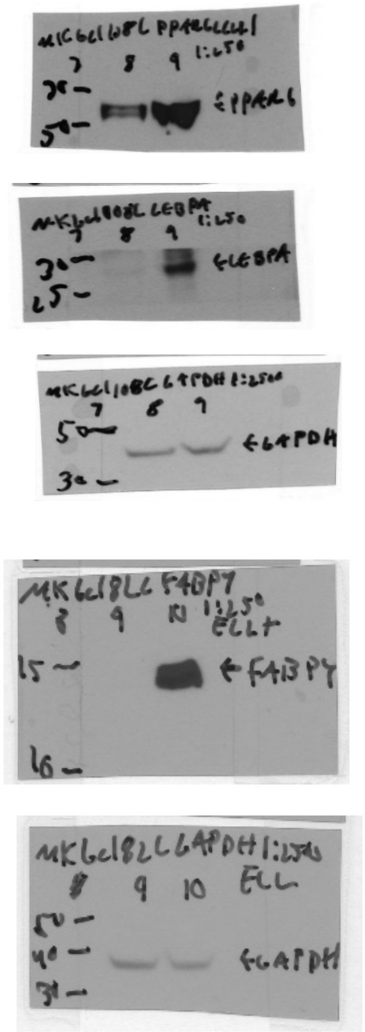

Supp. Fig. 6A (All Gels)	
**Lane 6**	Ladder
**Lane 7**	Scp-1 p000 Day 0 N1
**Lane 8**	Scp-1 p181a Day 0 N1
**Lane 9**	Scp-1 p000 Day 0 N2
**Lane 10**	Scp-1 p181a Day 0 N2

Supp. Fig. 6B (All Gels)	
**Lane 7 (Gel 108C) or Lane 8 (Gel 82)**	Ladder
**Lane 8 (Gel 108C) or Lane 9 (Gel 82)**	Scp-1 p000 Day 7
**Lane 9 (Gel 108C) or Lane 10 (Gel 82)**	Scp-1 p181a Day 7

Supplemental Figure 7

[A]

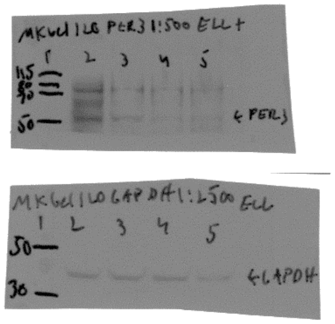

[B]

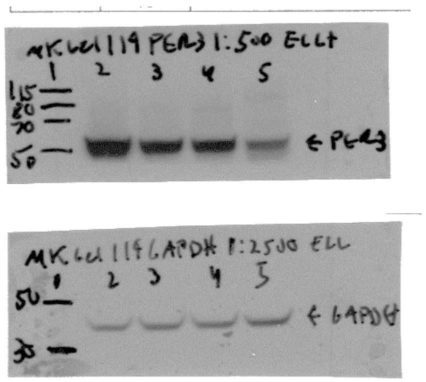

Supp. Fig. 7A	
**Lane 1**	Ladder
**Lane 2**	Scp-1 Adipo Day 0
**Lane 3**	Scp-1 Adipo Day 1
**Lane 4**	Scp-1 Adipo Day 3
**Lane 5**	Scp-1 Adipo Day 7

[C]

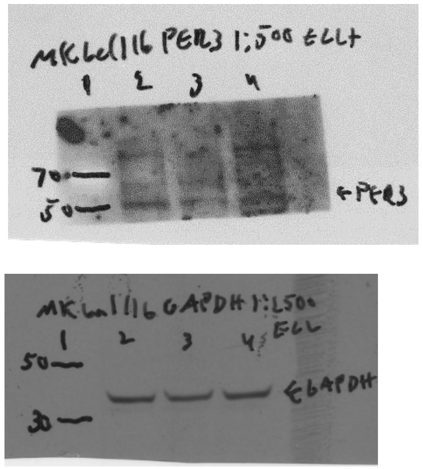

[D]

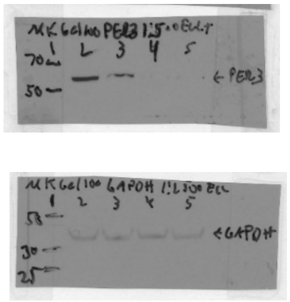

Supp. Fig. 7B	
**Lane 1**	Ladder
**Lane 2**	PMSC-1 Adipo Day 0
**Lane 3**	PMSC-1 Adipo Day 1
**Lane 4**	PMSC-1 Adipo Day 3
**Lane 5**	PMSC-1 Adipo Day 7

Supp. Fig. 7C	
**Lane 1**	Ladder
**Lane 2**	Scp-1 p000 Adipo Day 0
**Lane 3**	Scp-1 p181a Adipo Day 0
**Lane 4**	Scp-1 p181a-antimiR Adipo Day 0

Supp. Fig. 7D	
**Lane 1**	Ladder
**Lane 2**	PASC-1 p000 Adipo Day 0
**Lane 3**	PASC-1 p181a Adipo Day 0
**Lane 4**	PASC-1 p000 Adipo Day 7
**Lane 5**	PASC-1 p181a Adipo Day 7

Supplemental Figure 8

[A]

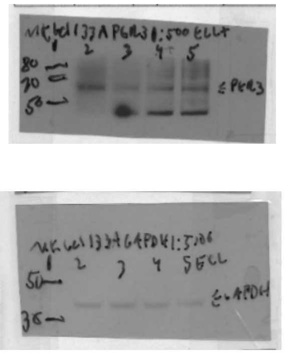

[B]

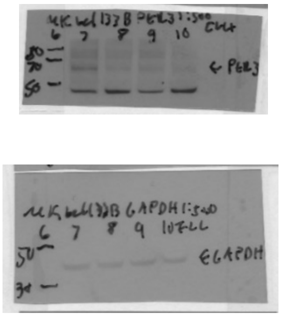

Supp. Fig. 8A	
**Lane 1**	Ladder
**Lane 2**	Scp-1 p000 Adipo Day 0
**Lane 3**	Scp-1 p181a Adipo Day 0
**Lane 4**	Scp-1 shPER3 #1 Adipo Day 0
**Lane 5**	Scp-1 shPER3 #2 Adipo Day 0

[C]

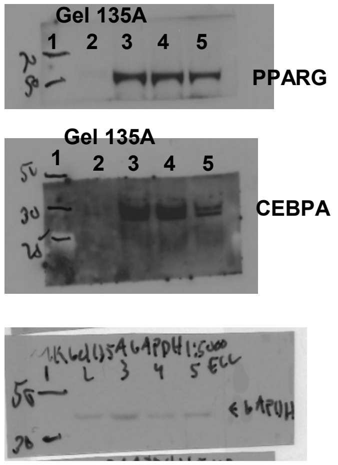

[D]

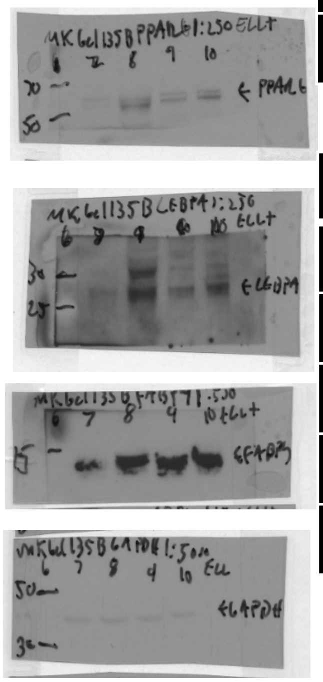

Supp. Fig. 8B	
**Lane 6**	Ladder
**Lane 7**	Scp-1 p000 Adipo Day 7
**Lane 8**	Scp-1 p181a Adipo Day 7
**Lane 9**	Scp-1 shPER3 #1 Adipo Day 7
**Lane 10**	Scp-1 shPER3 #2 Adipo Day 7

Supp. Fig. 8C	
**Lane 1**	Ladder
**Lane 2**	Scp-1 p000 Adipo Day 0
**Lane 3**	Scp-1 p181a Adipo Day 0
**Lane 4**	Scp-1 shPER3 #1 Adipo Day 0
**Lane 5**	Scp-1 shPER3 #2 Adipo Day 0

Supp. Fig. 8D	
**Lane 6**	Ladder
**Lane 7**	Scp-1 p000 Adipo Day 7
**Lane 8**	Scp-1 p181a Adipo Day 7
**Lane 9**	Scp-1 shPER3 #1 Adipo Day 7
**Lane 10**	Scp-1 shPER3 #2 Adipo Day 7

Supplemental Figure 9

[A]

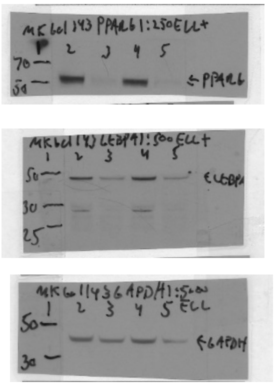

[B]

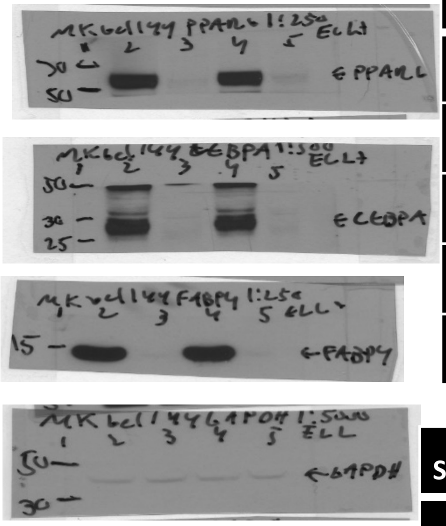

Supp. Fig. 9A	
**Lane 1**	Ladder
**Lane 2**	Scp-1 cmiR Adipo D0 N1
**Lane 3**	Scp-1 antimiR Adipo D0 N1
**Lane 4**	Scp-1 cmiR Adipo D0 N2
**Lane 5**	Scp-1 antimiR Adipo D0 N2

Supp. Fig. 9B	
**Lane 1**	Ladder
**Lane 2**	Scp-1 cmiR Adipo D21 N1
**Lane 3**	Scp-1 antimiR Adipo D21 N1
**Lane 4**	Scp-1 cmiR Adipo D21 N2
**Lane 5**	Scp-1 antimiR Adipo D21 N2

[C]

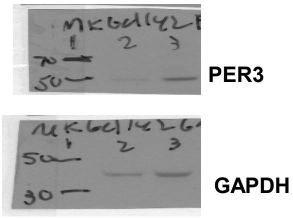

[D]

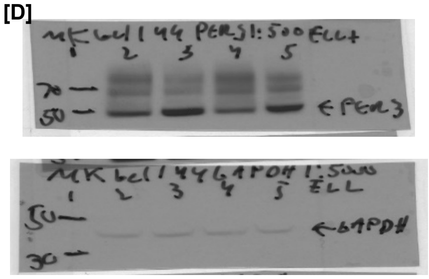

Supp. Fig. 9C	
**Lane 1**	Ladder
**Lane 2**	Scp-1 antimiR Adipo D0
**Lane 3**	Scp-1 cmiR Adipo D0

Supp. Fig. 9D	
**Lane 1**	Ladder
**Lane 2**	Scp-1 cmiR Adipo D21 N1
**Lane 3**	Scp-1 antimiR Adipo D21 N1
**Lane 4**	Scp-1 cmiR Adipo D21 N2
**Lane 5**	Scp-1 antimiR Adipo D21 N2

Supplemental Figure 10

[A]

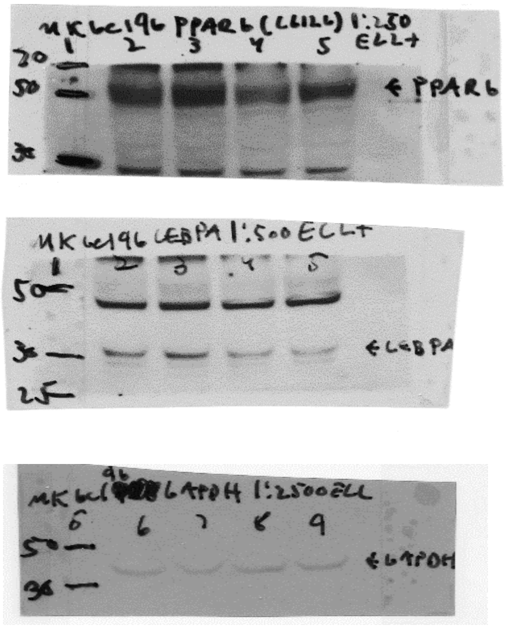

[B]

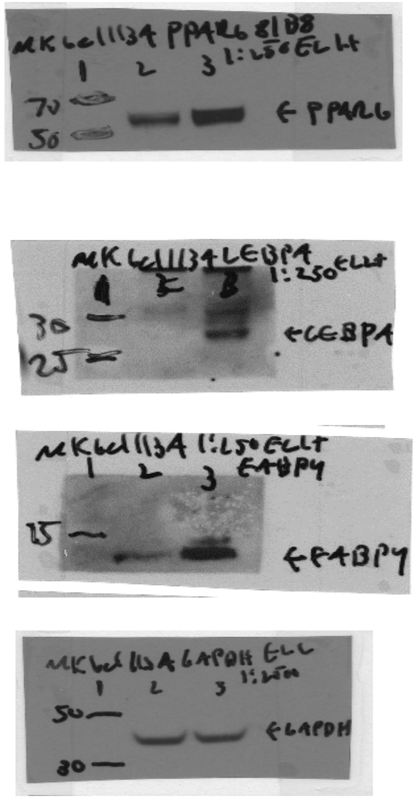

Supp. Fig. 10A	
**Lane 1**	Ladder
**Lane 2**	PASC-1 p000 Adipo Day 0 N1
**Lane 3**	PASC-1 p181a Adipo Day 0 N1
**Lane 4**	PASC-1 p000 Adipo Day 0 N2
**Lane 5**	PASC-1 p181a Adipo Day 0 N2

Supp. Fig. 10B	
**Lane 1**	Ladder
**Lane 2**	PASC-1 p000 Adipo Day 7 N1
**Lane 3**	PASC-1 p181a Adipo Day 7 N1

Supplemental Figure 11

[A]

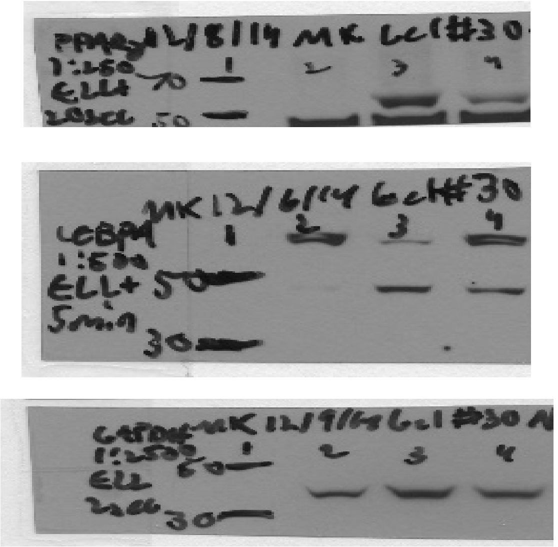

[B]

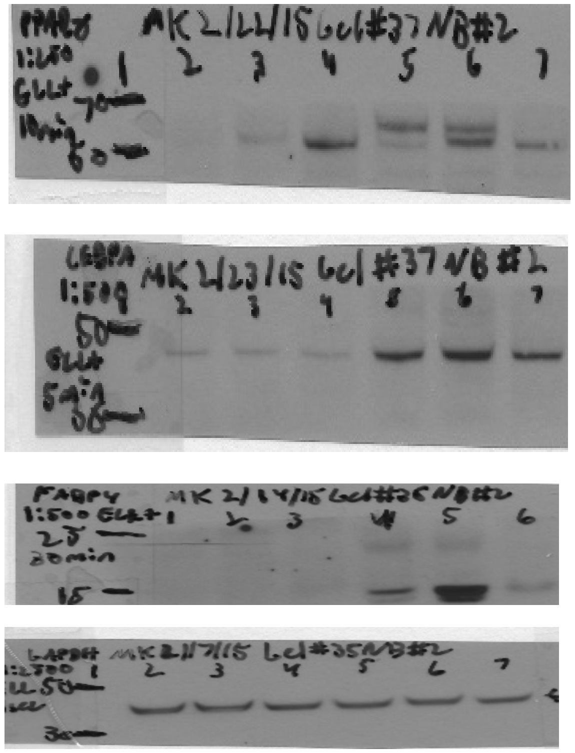

Supp. Fig. 11A	
Lane 1	Ladder
Lane 2	Scp-1 p000-shscram Adipo Day 0
Lane 3	Scp-1 p181a-shscram Adipo Day 0
Lane 4	Scp-1 p181a-shPPARG Adipo Day 0

Supp. Fig. 11B	
Lane 1	Ladder
Lane 5	Scp-1 p000-shscram Adipo Day 7
Lane 6	Scp-1 p181a-shscram Adipo Day 7
Lane 7	Scp-1 p181a-shPPARG Adipo Day 7

Supplemental Figure 12

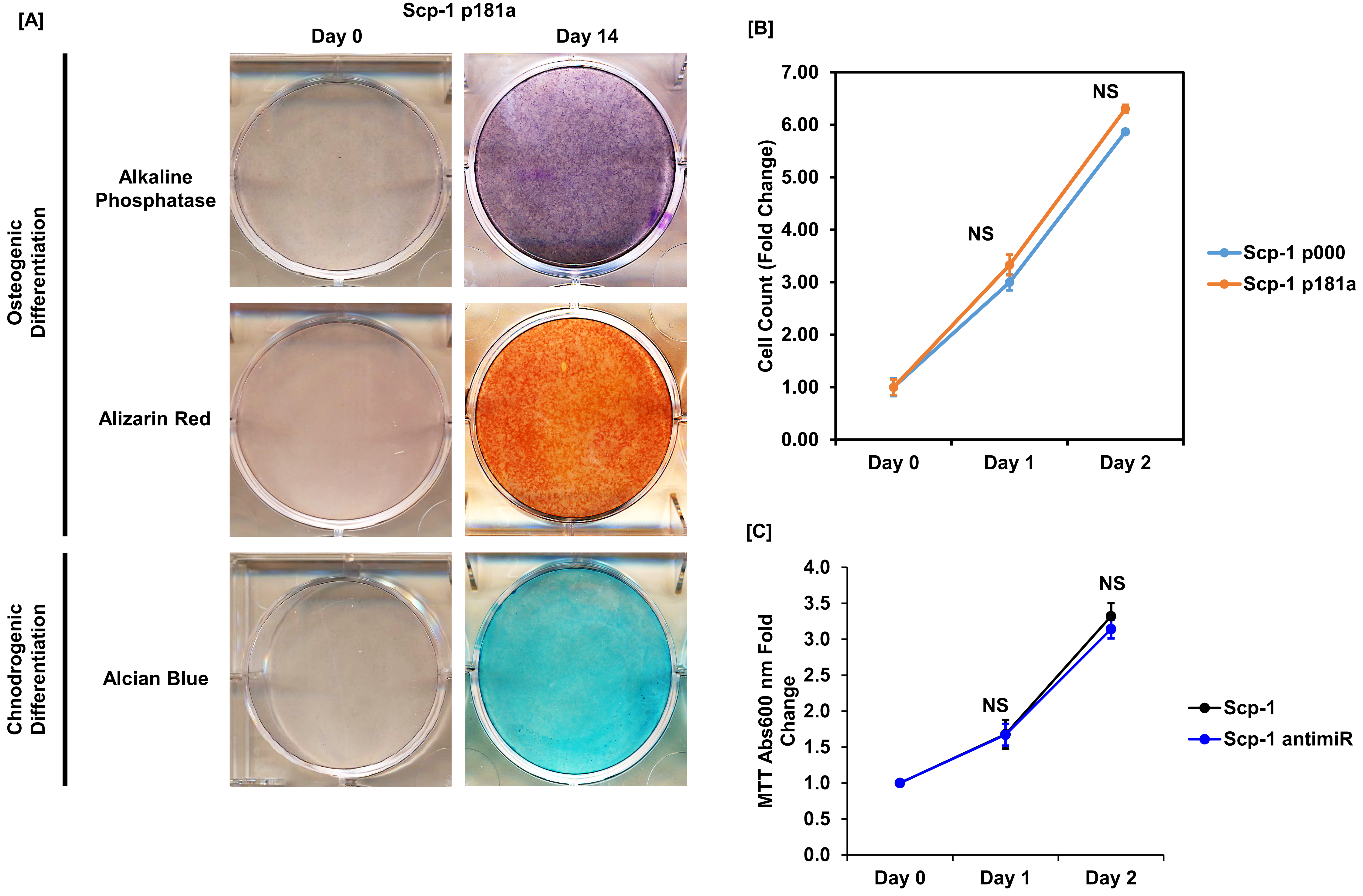

[D]

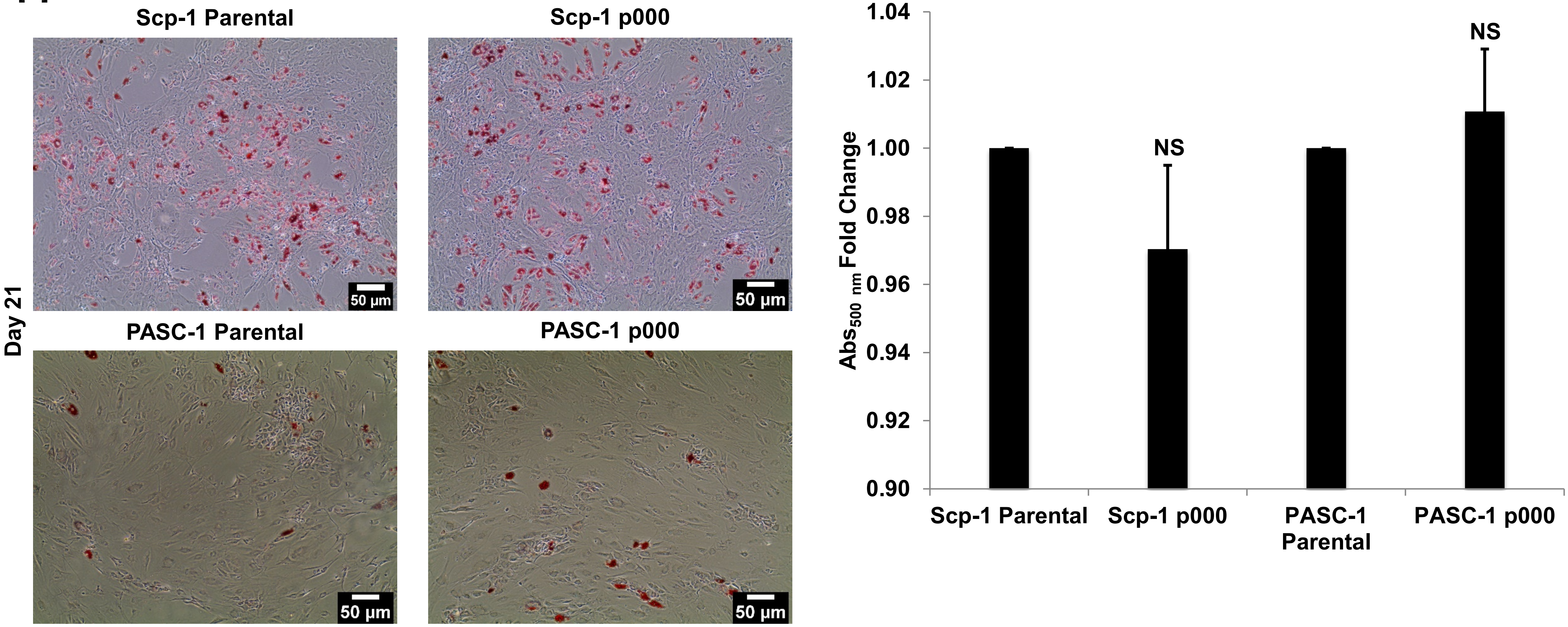
Scp-1 Parental
Scp-1 p000
PASC-1 Parental
PASC-1 p000
Day 21
50 µm
50 µm
50 µm
50 µm
$Abs_{500\ nm}$ Fold Change
1.04
1.02
1.00
0.98
0.96
0.94
0.92
0.90
NS
NS
Scp-1 Parental
Scp-1 p000
PASC-1 Parental
PASC-1 p000

miR-181a modulates circadian rhythm in immortalized bone marrow and adipose derived stromal cells and promotes differentiation through the regulation of PER3.

Matthew Knarr[1], Anil Belur Nagaraj[1], Lily J. Kwiatkowski[1], Analisa DiFeo[1]

[1]Case Comprehensive Cancer Center, Case Western Reserve University, Cleveland, OH 44106

Supplemental Figure 1: miR-181b expression does not correlate with adipogenesis in Scp-1 or PASC-1 cells A) Graph of miR-181b expression during adipogenic differentiation of parental Scp-1 cells at days 0,1,3,and 7. B) Graph of miR-181b expression during adipogenic differentiation of parental PASC-1 cells at days 0,1,3,and 7.

Supplemental Figure 2: Overexpression of miR-181a in PASC-1 MSCs increases adipogenesis

A) Oil red O staining for PASC-1 cells overexpressing either scramble vector (p000) or miR-181a (p181a) on day 14 of adipogenic differentiation with representative micrographs (top) and quantification of Oil red O staining for PASC-1 p000 and p181a cells at day 14 (bottom). B) Graph depicting increased expression of miR-181a at days 0 and 7 in PASC-1 p181a vs p000 cells. C) Graphs depicting increased mRNA expression of PPARG and CEBPA at day 0 and increased expression of adipogenesis target genes at day 7 in PASC-1 p181a vs p000 cells. D) Representative western blot images showing increased expression of CEBPA and PPARG in PASC-1 p181a vs p000 cells at day 0 with quantification (right) (Full blots are shown in Supplemental Figure 10A). Representative western blot images showing increased expression of CEBPA, PPARG, and

FABP4 at day 7 in PASC-1 p181a vs p000 cells with quantification (right) (Full blots are shown in Supplemental Figure 10B). Data are representative of 3 independent experiments. * is $p \leq 0.05$, ** is $p \leq 0.005$, *** is $p \leq 0.0005$

Supplemental Figure 3: Knockdown of PPARG decreases adipogenesis in Scp-1 p181a MSCs
A) Oil red O staining for Scp-1 cells overexpressing either scramble vector (p000) or miR-181a (p181a) with either a scramble shRNA (shscram) or shRNA against PPARG (shPPARG) on day 14 of adipogenic differentiation with representative micrographs (top) and quantification (right) (200X magnification). B) Graph depicting expression of miR-181a at days 0 and 7 in the Scp-1 p000-shscram, Scp-1 p181a-shscram, and Scp-1 p181a-shPPARG cells. C) Graphs depicting decreased expression of PPARG, CEBPA and terminal adipogenesis markers at days 0 and/or 7 in the Scp-1 p181a-shPPARG vs Scp-1 p181a-shscram cells. D & E) Representative western blot images showing decreased expression of CEBPA, PPARG and FABP4 at day 0 and/or day 7 in the Scp-1 p181a-shPPARG vs Scp-1 p181a-shscram cells with quantification (Full blots are shown in Supplemental Figure 11A-B). * is $p \leq 0.05$, ** is $p \leq 0.005$, *** is $p \leq 0.0005$.

Supplemental Figure 4: Knockdown of miR-181a decreases adipogenesis in Scp-1 p181a MSCs

A) Oil red O staining for Scp-1 cells overexpressing either scramble vector (p000), the combination of miR-181a (p181a) and scramble antagomiR (cmiR), or the combination of miR-181a (p181a) and miR-181a antagomiR (antimiR) on day 14 of adipogenic differentiation with representative micrographs (top) and quantification of Oil red O staining for Scp-1 p000, Scp-1 p181a-cmiR, and Scp-1 p181a-antimiR cells at day 14 (right) (200X magnification). B) Graphs depicting decreased expression of miR-181a, CEBPA, and PPARG at day 7 in Scp-1 p181a-cmiR vs p181a-antimiR cells. C) Graphs showing decreased expression of CEBPA, PPARG, and

terminal adipogenesis markers at day 7 in Scp-1 p181a-cmiR vs p181a-antimiR cells. * is $p \leq 0.05$, ** is $p \leq 0.005$, *** is $p \leq 0.0005$

Supplemental Figure 5: Full western blots for Figure 1C and 1D

A) Full western blots for Figure 1C showing uncropped images for each target along with respective loading control images for each gel. B) Full western blots for Figure 1D showing uncropped images for each target along with respective loading control images for each gel. Lane orders for each subfigure are shown in the list below

Supp. Fig. 5A (All Gels)	
Lane 1	Ladder
Lane 2	Scp-1 Adipo Day 0
Lane 3	Scp-1 Adipo Day 1
Lane 4	Scp-1 Adipo Day 3
Lane 5	Scp-1 Adipo Day 7

Supp. Fig. 5B (All Gels)	
Lane 1	Ladder
Lane 2	PASC-1 Adipo Day 0
Lane 3	PASC-1 Adipo Day 1
Lane 4	PASC-1 Adipo Day 3
Lane 5	PASC-1 Adipo Day 7

Supplemental Figure 6: Full western blots for Figure 2D and 2E

A) Full western blots for Figure 2D showing uncropped images for each target along with respective loading control images for each gel. B) Full western blots for Figure 2E showing uncropped images for each target along with respective loading control images for each gel. Lane orders for each subfigure are shown in the list below

Supp. Fig. 6A (All Gels)	
Lane 6	Ladder
Lane 7	Scp-1 p000 Day 0 N1
Lane 8	Scp-1 p181a Day 0 N1
Lane 9	Scp-1 p000 Day 0 N2
Lane 10	Scp-1 p181a Day 0 N2

Supp. Fig. 6B (All Gels)	
Lane 7 (Gel 108C) or Lane 8 (Gel 82)	Ladder
Lane 8 (Gel 108C) or Lane 9 (Gel 82)	Scp-1 p000 Day 7
Lane 9 (Gel 108C) or Lane 10 (Gel 82)	Scp-1 p181a Day 7

Supplemental Figure 7: Full western blots for Figure 3C, 3E, and 3G

A) Full western blots for Figure 3C showing uncropped images for each target along with respective loading control images for each gel. B) Full western blots for Figure 3E showing uncropped images for each target along with respective loading control images for each gel. C-D) Full western blots for Figure 3G showing uncropped images for each target along with respective loading control images for each gel. Lane orders for each subfigure are shown in the list below

Supp. Fig. 7A	
Lane 1	Ladder
Lane 2	Scp-1 Adipo Day 0
Lane 3	Scp-1 Adipo Day 1
Lane 4	Scp-1 Adipo Day 3
Lane 5	Scp-1 Adipo Day 7

Supp. Fig. 7B	
Lane 1	Ladder
Lane 2	PASC-1 Adipo Day 0
Lane 3	PASC-1 Adipo Day 1
Lane 4	PASC-1 Adipo Day 3
Lane 5	PASC-1 Adipo Day 7

Supp. Fig. 7C	
Lane 1	Ladder
Lane 2	Scp-1 p000 Adipo Day 0
Lane 3	Scp-1 p181a Adipo Day 0
Lane 4	Scp-1 p181a-antimiR Adipo Day 0

Supp. Fig. 7D	
Lane 1	Ladder

Lane 2	PASC-1 p000 Adipo Day 0
Lane 3	PASC-1 p181a Adipo Day 0
Lane 4	PASC-1 p000 Adipo Day 7
Lane 5	PASC-1 p181a Adipo Day 7

Supplemental Figure 8: Full western blots for Figure 4C, 4F, and 4G

A & B) Full western blots for Figure 4C showing uncropped images for each target along with respective loading control images for each gel. C) Full western blots for Figure 4F showing uncropped images for each target along with respective loading control images for each gel. D) Full western blots for Figure 4G showing uncropped images for each target along with respective loading control images for each gel. Lane orders for each subfigure are shown in the list below

Supp. Fig. 8A	
Lane 1	Ladder
Lane 2	Scp-1 p000 Adipo Day 0
Lane 3	Scp-1 p181a Adipo Day 0
Lane 4	Scp-1 shPER3 #1 Adipo Day 0
Lane 5	Scp-1 shPER3 #2 Adipo Day 0

Supp. Fig. 8B	
Lane 6	Ladder
Lane 7	Scp-1 p000 Adipo Day 7
Lane 8	Scp-1 p181a Adipo Day 7

Lane 9	Scp-1 shPER3 #1 Adipo Day 7
Lane 10	Scp-1 shPER3 #2 Adipo Day 7

Supp. Fig. 8C	
Lane 1	Ladder
Lane 2	Scp-1 p000 Adipo Day 0
Lane 3	Scp-1 p181a Adipo Day 0
Lane 4	Scp-1 shPER3 #1 Adipo Day 0
Lane 5	Scp-1 shPER3 #2 Adipo Day 0

Supp. Fig. 8D	
Lane 6	Ladder
Lane 7	Scp-1 p000 Adipo Day 7
Lane 8	Scp-1 p181a Adipo Day 7
Lane 9	Scp-1 shPER3 #1 Adipo Day 7
Lane 10	Scp-1 shPER3 #2 Adipo Day 7

Supplemental Figure 9: Full western blots for Figure 5D, 5E, and 5G

A) Full western blots for Figure 5D showing uncropped images for each target along with respective loading control images for each gel. B) Full western blots for Figure 5E showing uncropped images for each target along with respective loading control images for each gel. C-D) Full western blots for Figure 5G showing uncropped images for each target along with respective

loading control images for each gel. Lane orders for each subfigure are shown in the list below.

*Blots in Supp Figure 8C are flipped horizontally in Figure 5.

Supp. Fig. 9A	
Lane 1	Ladder
Lane 2	Scp-1 cmiR Adipo D0 N1
Lane 3	Scp-1 antimiR Adipo D0 N1
Lane 4	Scp-1 cmiR Adipo D0 N2
Lane 5	Scp-1 antimiR Adipo D0 N2

Supp. Fig. 9B	
Lane 1	Ladder
Lane 2	Scp-1 cmiR Adipo D21 N1
Lane 3	Scp-1 antimiR Adipo D21 N1
Lane 4	Scp-1 cmiR Adipo D21 N2
Lane 5	Scp-1 antimiR Adipo D21 N2

Supp. Fig. 9C	
Lane 1	Ladder
Lane 2	Scp-1 antimiR Adipo D0
Lane 3	Scp-1 cmiR Adipo D0

Supp. Fig. 9D	
Lane 1	Ladder
Lane 2	Scp-1 cmiR Adipo D21 N1
Lane 3	Scp-1 antimiR Adipo D21 N1
Lane 4	Scp-1 cmiR Adipo D21 N2
Lane 5	Scp-1 antimiR Adipo D21 N2

Supplemental Figure 10: Full western blots for Supp Figure 1D and 1E

A) Full western blots for Supp Figure 1D showing uncropped images for each target along with respective loading control images for each gel. B) Full western blots for Supp Figure 1E showing uncropped images for each target along with respective loading control images for each gel. Lane orders for each subfigure are shown in the list below.

Supp. Fig. 10A	
Lane 1	Ladder
Lane 2	PASC-1 p000 Adipo Day 0 N1
Lane 3	PASC-1 p181a Adipo Day 0 N1
Lane 4	PASC-1 p000 Adipo Day 0 N2
Lane 5	PASC-1 p181a Adipo Day 0 N2

Supp. Fig. 10B	
Lane 1	Ladder

Lane 2	PASC-1 p000 Adipo Day 7 N1
Lane 3	PASC-1 p181a Adipo Day 7 N1

Supplemental Figure 11: Full western blots for Supp Figure 2D and 2E

A) Full western blots for Supp Figure 2D showing uncropped images for each target along with respective loading control images for each gel. B) Full western blots for Supp Figure 2E showing uncropped images for each target along with respective loading control images for each gel. Lane orders for each subfigure are shown in the list below. *All blots in Supp Figure 10B are from Gel 35 (top 2 mislabled)

Supp. Fig. 11A	
Lane 1	Ladder
Lane 2	Scp-1 p000-shscram Adipo Day 0
Lane 3	Scp-1 p181a-shscram Adipo Day 0
Lane 4	Scp-1 p181a-shPPARG Adipo Day 0

Supp. Fig. 11B	
Lane 1	Ladder
Lane 5	Scp-1 p000-shscram Adipo Day 7
Lane 6	Scp-1 p181a-shscram Adipo Day 7
Lane 7	Scp-1 p181a-shPPARG Adipo Day 7

Supplemental Figure 12: Scp-1 p181a cells maintain multipotency and do not differ from Scp-1 p000 cells in terms of proliferation

A) Plate scan images of Scp-1 p181a cells at day 0 or day 14 that had undergone either osteogenic or chondrogenic differentiation. Both osteogenic and chondrogenic differentiation were performed using either StemPro™ Osteogenesis Differentiation Kit or StemPro™ Chondrogenesis Differentiation Kit according to the manufacturer's instructions respectively. Cells were stained for alkaline phosphatase activity or using alizarin red (Ca^{+2} deposits) to measure osteogenesis. Cells were stained with alcian blue to measure chondrogenesis. B) Cell counting of Scp-1 p000 and Scp-1 p181a at 24 hr (Day 0), 48 hr (Day 1), and 96 hr (Day 2) after plating. Proliferation is expressed as fold change normalized to day 0 for each cell line. Results are representative of 3 biological replicates. C) MTT assay of Scp-1 cmiR and hmiR cells at 24hr (Day 0), 48 hr (Day 1), and 96 hr (Day 2) after plating. Cell viability is expressed as fold change normalized to day 0 for each cell line. Results are representative of 3 biological replicates. D) Oil red O staining of Scp-1 and PASC-1 parental and p000 cells at day 21 post adipogenic induction with quantification (right). Images and quantification are representative of 3 biological replicates.
